# Lipid-tail modification of the antimicrobial peptide P7 enhances membrane disruption and antibacterial activity against *Klebsiella pneumoniae*

**DOI:** 10.7717/peerj.21717

**Published:** 2026-10-07

**Authors:** Warintorn Thammathaporn, Natthaporn Klubthawee Aiemwichan, Ratchaneewan Aunpad

**Affiliations:** 1 Graduate Program in Biomedical Sciences, Faculty of Allied Health Sciences, Thammasat University, Khlong Luang, Pathum Thani, Thailand; 2 Department of Medical Technology, Faculty of Allied Health Sciences, Thammasat University, Khlong Luang, Pathum Thani, Thailand

**Keywords:** Antimicrobial peptide, P7, Lipidation, *Klebsiella pneumoniae*, ESKAPE, Infectious disease

## Abstract

Antimicrobial peptides (AMPs) are short, host-defense peptides with broad-spectrum antibacterial activity and a low propensity for inducing resistance, positioning them as promising alternatives to conventional antibiotics. The α-helical peptide P7 (KIAKRIWKILRR) previously demonstrated potent antibacterial activity against drug-resistant *Salmonella enterica* serovar Typhimurium with minimal toxicity. However, its efficacy against ESKAPE pathogens (*Enterococcus faecium*, *Staphylococcus aureus*, *Klebsiella pneumoniae*, *Acinetobacter baumannii*, *Pseudomonas aeruginosa*, and *Enterobacter* spp.) has not been characterized. In this study, we evaluated P7 and its N-terminal lipidated analogs conjugated with octanoic (C8), pelargonic (C9), and capric (C10) acids against ESKAPE organisms. Lipidation significantly enhanced antibacterial activity, particularly against *K. pneumoniae*, and promoted α-helical conformations in membrane-mimetic environments. Lipidated peptides also demonstrated improved membrane-permeabilizing and depolarizing capabilities. However, lipidation did not confer enhanced proteolytic stability. C8-P7 exhibited a more favorable activity profile among the analogs. The lipidated variants showed a limited therapeutic index, indicating that further structural optimization is required to improve their selectivity. Flow cytometry and electron microscopy confirmed membrane disruption as the primary mechanism of antibacterial action. Furthermore, additive effects were observed when C8-P7 was combined with gentamicin or ciprofloxacin, as indicated by fractional inhibitory concentration index. These findings highlighted the potential of lipid-tail engineering to enhance antimicrobial potency and membrane-disruptive capacity, offering a foundation for the development of potent lead compounds against multidrug-resistant *K. pneumoniae* and other ESKAPE pathogens.

## Introduction

One of the major challenges in controlling hospital-acquired infections (HAIs) worldwide is the increasing incidence of multidrug-resistant ESKAPE pathogens, a group consisting of *Enterococcus faecium*, *Staphylococcus aureus*, *Klebsiella pneumoniae*, *Acinetobacter baumannii*, *Pseudomonas aeruginosa*, and *Enterobacter* spp. (Mulani et al., 2019). These organisms are particularly dangerous for critically ill and immunocompromised individuals, owing to their robust resistance mechanisms and capacity to evade conventional antimicrobial therapies (Santajit & Indrawattana, 2016). According to the World Health Organization (WHO), ESKAPE pathogens are priority targets for the development of novel antimicrobial agents due to their clinical importance (De Oliveira et al., 2020). The growing threat of antibiotic resistance posed by these pathogens underscores an urgent need for coordinated global efforts to develop effective alternative therapies (Chinemerem Nwobodo et al., 2022).

Antimicrobial peptides (AMPs) have been recognized as viable alternatives to traditional antibiotics for combating multidrug-resistant pathogens (Moretta et al., 2021). Because AMPs target multiple cellular components, they are less likely to induce resistance and so remain effective against multidrug-resistant bacteria (Xuan et al., 2023). All living organisms naturally produce small bioactive peptides as part of their innate immune systems (Luo & Song, 2021). AMPs demonstrate broad-spectrum efficacy, directly targeting bacteria, viruses, fungi, yeasts, and even cancer cells (Lei et al., 2019). Structurally, AMPs are typically amphipathic, comprising cationic residues that confer a hydrophilic surface on one side of the molecule and hydrophobic groups that form the opposite face. Due to their amphipathic nature and overall positive charge, AMPs preferentially interact with negatively charged bacterial membranes, as an initial step that leads to cell death, which is one of their mechanisms of action (Szymczak et al., 2023).

Despite their numerous potential advantages, the translation of AMPs into the clinic remains limited by several challenges, most notably poor stability, susceptibility to proteolytic degradation, and tendency to exhibit lower direct antibacterial activity compared to antibiotics (Rima et al., 2021). These factors restrict their therapeutic efficacy and pharmacological development. To overcome these challenges, lipidation (achieved by conjugating AMPs with fatty acids) has emerged as an effective strategy. This modification primarily enhances membrane interactions and hydrophobic anchoring, thereby significantly increasing antibacterial potency (Rounds & Straus, 2020). The antibacterial activity of peptide conjugates is influenced by the length of the attached fatty acid chain (Desbois & Smith, 2010). Short-chain fatty acids often exhibit suboptimal interactions with bacterial membranes, resulting in reduced efficacy. In contrast, medium-chain fatty acids tend to enhance membrane affinity, consistently yielding low minimum inhibitory concentrations (MICs) and improved antibacterial activity. Conversely, long-chain fatty acids tend to form aggregates, which can hinder membrane interaction and diminish antimicrobial activity, as reflected by elevated MIC values (Grimsey et al., 2020). Previous studies suggest that fatty acid chains ranging from C8–C12 represent the optimal length for peptide lipidation, as they enhance membrane insertion and promote secondary structure stabilization (Bellavita et al., 2023; Zhong et al., 2020). Such physicochemical features contribute to increased antibacterial selectivity and potency.

In this study, we utilized P7, a previously characterized 12-mer cationic antimicrobial peptide, as a structural template. Originally designed using a template-modified strategy based on the cathelicidin family, P7 is characterized by a robust α-helical conformation and high thermostability. Previous investigations demonstrated that P7 exerts potent membrane-permeabilizing activity against drug-resistant *Salmonella enterica* serovar Typhimurium and its monophasic variants, while maintaining negligible hemolytic activity (Klubthawee & Aunpad, 2020). Despite these favorable properties, further enhancement is required to maximize its therapeutic potential against the more challenging ESKAPE panel. Consequently, we employed a lipidation strategy, conjugating P7 with medium-chain fatty acids specifically octanoic acid (C8), pelargonic acid (C9), and capric acid (C10) to evaluate the impact of lipid modification on its antimicrobial potency and proteolytic stability. The resulting lipidated P7 variants were subjected to a comprehensive evaluation against clinically relevant ESKAPE pathogens, including assessments of cytotoxicity, hemolytic activity, stability in physiological environments, and potential synergistic interactions with conventional antibiotics. Our findings reveal that while lipidation significantly enhances antimicrobial efficacy, it also impacts the peptides’ safety profile, highlighting a critical balance between potency and host cell toxicity that must be addressed to improve their overall therapeutic index.

## Materials and Methods

### Peptide synthesis and preparation

Peptide synthesis was carried out by China Peptides (Shanghai, China) using solid-phase synthesis with 9-fluorenylmethoxycarbonyl (Fmoc) chemistry. This included the parent peptide P7 and N-terminal fatty acid conjugates of varying carbon chain lengths, namely octanoic (C8-P7), pelargonic (C9-P7), and capric (C10-P7) acids (Table 1). Following synthesis, the peptides were purified by reversed-phase high-performance liquid chromatography (RP-HPLC), yielding trifluoroacetate salts with a purity exceeding 95%. Peptide masses were confirmed by electrospray ionization mass spectrometry (ESI-MS). The HPLC chromatograms and ESI-MS spectra for all tested peptides have been provided in the Figs. S1 and S2. Prior to use, all peptides were reconstituted in phosphate-buffered saline (PBS) (Caisson Labs, Smithfield, UT, USA) at a stock concentration of 10 mg/mL and stored at −20 °C.

**Table 1:** Physicochemical properties of peptides and their sequences.

Peptide	Peptide sequence	Theoretical MW (Da)	Calculated m/z [M + 4H]^4+^	Found m/z [M + 4H]^4+a^	T_R_ (min)^b^
P7	KIAKRIWKILRR-NH_2_	1,580.05	396.01	396.09	7.656
C8-P7	Octanoic acid-KIAKRIWKILRR-NH_2_	1,706.15	427.54	427.46	10.224
C9-P7	Pelargonic acid-KIAKRIWKILRR-NH_2_	1,720.17	431.04	431.07	10.434
C10-P7	Capric acid-KIAKRIWKILRR-NH_2_	1,734.18	434.55	434.79	11.984

**Notes:**

a Observed m/z value of the [M + 4H]^4+^ charge state determined by ESI-MS analysis.

b T_R_, retention time was determined by RP-HPLC.

### Bacterial strains and growth conditions

Bacterial strains representing the ESKAPE pathogens were obtained from the ATCC and DMST collections, including *E. faecium* ATCC 700221, *S. aureus* ATCC 25923, *K. pneumoniae* ATCC 27736, *A. baumannii* DMST 43250, *P. aeruginosa* DMST 15501, and *E. cloacae* DMST 553. All strains were cultured in Tryptic Soy Broth (TSB; BD Difco, Franklin Lakes, NJ, USA), except for E. *faecium* ATCC 700221, which was grown in Brain Heart Infusion (BHI) broth (Thermo Scientific, Waltham, MA, USA). Each culture was incubated at 37 °C with continuous shaking at 220 rpm for 16 to 18 h.

### Antibacterial assay

Minimum inhibitory concentrations (MICs) of the peptides were determined using a broth microdilution method, modified from the National Committee for Clinical Laboratory Standards (NCCLS) guidelines (Steinberg et al., 1997). Briefly, two-fold serial dilutions of peptides (0.98–250 μg/mL) were incubated with bacterial suspensions (approximately 10^7^ CFU/mL; OD_620_ = 0.05) in Mueller-Hinton broth (MHB; BD Difco, Franklin Lakes, NJ, USA) at 37 °C with shaking at 220 rpm for 24 h. The MIC was defined as the lowest peptide concentration that inhibited visible bacterial growth (Parvekar et al., 2020). The minimum bactericidal concentration (MBC) was determined using a colony count assay by plating aliquots from clear wells onto tryptic soy agar (TSA; BD Difco, Franklin Lakes, NJ, USA) and incubating overnight at 37 °C (CLSI, 2024). MBC was defined as the lowest concentration with no visible colony formation (Parvekar et al., 2020). Inoculated broth without peptides served as a negative control. Experiments were conducted in three independent biological replicates.

### Peptide stability in the presence of proteolytic enzymes

Peptide stability was assessed by measuring residual antibacterial activity following treatment with proteolytic enzymes as previously described (Xu et al., 2014; Zou et al., 2025) with minor modifications. To simulate gastric acidity, 0.01 M HCl (pH 2.0) was employed as the buffer for pepsin (Sigma-Aldrich, St. Louis, MO, USA), while PBS (pH 7.4) was used for trypsin (AppliChem, Darmstadt, Germany) to represent neutral physiological environments. Briefly, peptides were prepared in the respective buffers (0.01 M HCl for pepsin and PBS for trypsin) and pre-incubated with the enzymes at 37 °C for 1 h. The enzyme stock solutions were freshly prepared and the final concentrations in the reaction mixture were calculated to achieve 1 mg/mL. Following incubation, the enzymatic reactions were terminated: pepsin-treated samples were neutralized to pH 7.0 by the addition of 0.1 M NaOH, while trypsin-treated samples were inactivated by heating at 60 °C for 15 min. The residual antimicrobial activity of the neutralized mixtures was then determined *via* the antibacterial assay (MIC determination) against *K. pneumoniae* ATCC 27736 as described above. All experiments were conducted in three independent biological replicates.

### Circular dichroism analysis

The secondary structures of peptide derivatives were analyzed using a Jasco-815 spectropolarimeter (Jasco, Tokyo, Japan) operated under a nitrogen atmosphere at 25 °C, using a quartz cuvette with a 0.1 cm pathlength (Klubthawee et al., 2020). Briefly, peptides were diluted to 0.2 mg/mL in various solvents to mimic several different biological environments: 50% (v/v) 2,2,2-trifluoroethanol (TFE; Sigma-Aldrich, St. Louis, MO, USA) to mimic hydrophobic microbial membrane environments; 30 mM sodium dodecyl sulfate (SDS) micelles (Sigma-Aldrich, St. Louis, MO, USA) to simulate negatively-charged prokaryotic membranes; phosphate-buffered saline (PBS) to represent aqueous physiological environments. Circular dichroism (CD) spectra in the wavelength range of 190–260 nm were recorded over at least three scans per condition, at a scanning speed of 10 nm/min. The mean residue ellipticity (MRE) was calculated from the CD data using the following formula:

$\theta_{\rm M} = (\theta_{\rm obs} \times {\rm M}_{\rm RW}) / (10 \times {\rm c} \times {\rm l}).$

In the equation, θ_M_ denotes the mean residue ellipticity (deg. M^–1^ m^–1^), calculated using the observed ellipticity (θ_obs_) corrected for buffer contribution at a specific wavelength (mdeg), the mean residue weight (M_RW_; total molecular weight divided by the number of amino acids), the peptide concentration (c, in mg/mL), and the path length (l, in cm).

### Hemolytic assay

The hemolytic activity of peptide derivatives was determined using human red blood cells (hRBCs) by quantifying hemoglobin released following peptide treatment (Klubthawee et al., 2020). The hRBCs from healthy volunteers were isolated from freshly collected blood in a heparin-coated polycarbonate tube. The sample was centrifuged at 2,000 × g for 5 min, washed at least three times with PBS, and resuspended to 2% (v/v). The suspension was mixed with two-fold serial dilutions of peptides (0.98–250 μg/mL) in PBS and incubated at 37 °C for 1 h. Samples were then centrifuged at 2,000 × g for 10 min, and supernatants were transferred to a 96-well plate. Absorbance at 405 nm was measured using a Multiskan SkyHigh microplate reader (Thermo Scientific, Waltham, MA, USA). Triton X-100 (0.1%) and PBS served as positive (100%) and negative (0%) hemolysis controls, respectively. All experiments were conducted in three independent biological replicates. Hemolysis percentage was calculated using the following formula:

${\rm\%} \;{\rm Hemolysis} = ({\rm OD}_{\rm Sample}- {\rm OD}_{\rm Blank}) / ({\rm OD}_{\rm Triton\; X\mathrm{-}100} - {\rm OD}_{\rm Blank}) \times 100.$

All experiments involving human subjects were conducted with approval from the Institutional Ethics Committee of Thammasat University (COA No. 009/2568) and followed the University’s ethical guidelines. Written informed consent was obtained from all participants prior to sample collection.

### Flow cytometry analysis

The propidium iodide (PI), a membrane-impermeable nucleic acid intercalator, and bis-(1,3-dibutylbarbituric acid) trimethine oxonol (BOX), a membrane potential-sensitive dye (both from Sigma-Aldrich, St. Louis, MO, USA), were employed to evaluate peptide-induced membrane permeabilization and depolarization (Rabanal et al., 2015). Briefly, peptide derivatives at 1× MIC were added to bacterial suspensions (OD_620_ = 0.05) in MHB and incubated at 37 °C with shaking at 220 rpm for 0.5, 1, 2, and 4 h. After incubation, bacterial cells were centrifuged at 10,000 × g for 10 min, resuspended, and stained with a combination of PI and BOX. Prior to measurement, samples were incubated at room temperature in the dark for 15 min. Bacterial cells treated with melittin served as positive controls, while untreated cells served as negative controls. A BD Accuri™ C6 Plus Flow Cytometer (BD, Franklin Lakes, NJ, USA) with 488 nm laser excitation was used to collect fluorescence data. Signals were assessed on logarithmic scales for red fluorescence (585/42 nm; PI), green fluorescence (530/30 nm; BOX), side scatter (SS), and forward scatter (FS). A minimum of 50,000 events were recorded per sample. Each experiment was conducted in three independent biological replicates, and data analyses were conducted using FlowJo™ software.

### *In vitro* cytotoxicity assay

Viability of L929 mouse fibroblasts (NCTC clone 929) was determined using the 3-(4,5-dimethylthiazol)-2,5-diphenyl-tetrazolium bromide (MTT; Sigma-Aldrich, St. Louis, MO, USA) assay as previously described (Zhang et al., 2016) with some modifications. Briefly, L929 cells were cultured in Dulbecco’s Modified Eagle Medium (DMEM; Gibco, Grand Island, NY, USA) supplemented with 10% fetal bovine serum (FBS; Gibco, Grand Island, NY, USA) and 1% (v/v) of penicillin-streptomycin and incubated at 37 °C in a humidified atmosphere containing 5% CO_2_. L929 cells were seeded in 96-well plates at a density of approximately 3 × 10^4^ cells/well and then treated with the peptide at a dilution in the range of 0.98–250 μg/mL, followed by incubation for an additional 24 h under the same conditions. MTT solution (final concentration of 0.5 mg/mL) was added to each well and incubated for 3 h at 37 °C with 5% CO_2_. The supernatants were discarded. Dimethyl sulfoxide (DMSO) was then added to dissolve the formazan crystals, and absorbance was measured at 570 nm using a Multiskan FC microplate reader. The positive control consisted of medium with cells, while the negative control was medium alone. Experiments were conducted in three independent biological replicates, and the following formula was used to calculate the percentage of cell viability:

$\%\; {\rm Cell\; viability} = ({\rm OD}_{\rm 570\; of\; treated\; sample} - {\rm OD}_{\rm Blank}) / ({\rm OD}_{\rm 570\;of\; untreated\; sample} - {\rm OD}_{\rm Blank}) \times 100.$

### Time-kill kinetics assay

Bactericidal kinetics were assessed to evaluate the killing efficacy of the lipidated P7 peptide on bacteria at different exposure durations, as previously described (Klubthawee et al., 2020). Briefly, bacterial suspensions (OD_620_ = 0.05) were treated with peptide at MIC in MHB and incubated at 37 °C with shaking (220 rpm). Aliquots were collected at 0, 0.5, 1, 2, 4, 6, and 24 h, then serially diluted ten-fold in PBS and plated onto TSA. Colony-forming units (CFU/mL) were quantified after incubating the plates at 37 °C for 24 h. Negative controls consisted of bacterial suspensions treated with PBS only and were included in each experimental run. Results are presented as the mean of three independent replicates.

### Transmission electron microscopy

Structural alterations and membrane damage in bacterial cells following treatment with lipidated P7 peptide were examined using transmission electron microscopy (TEM), as previously described (Klubthawee & Aunpad, 2020) with minor modifications. Briefly, bacterial suspensions (OD_620_ = 0.05) were treated with lipidated peptide at 1× MIC for 2 h at 37 °C. After centrifugation at 10,000 × g for 10 min, bacterial pellets were fixed overnight at 4 °C in 2.5% glutaraldehyde and then post-fixed in 1% osmium tetroxide (OsO_4_) for 1 h. Cells were dehydrated through a graded ethanol series (50%, 70%, 90%, and 100%) and rinsed with absolute acetone. Infiltration was carried out using a 1:1 mixture of absolute acetone and epoxy resin, followed by immersion in a 1:3 mixture for 1 h. Ultrathin sections were prepared using an ultramicrotome (Leica EM UC7; Leica, Wien, Austria), stained with uranyl acetate and lead citrate, and examined by TEM (Hitachi HT7700; Hitachi, Tokyo, Japan). Bacterial cells without treatment were used as negative controls.

### Scanning electron microscopy

Changes in bacterial surface morphology induced by lipidated P7 peptide were analyzed using scanning electron microscopy (SEM), as previously described (Lv et al., 2014) with minor modifications. Bacterial suspensions were prepared under the same conditions as those used for TEM. Briefly, pelleted cells were collected on membrane filters, fixed overnight at 4 °C in 2.5% (v/v) glutaraldehyde, followed by post-fixation with 1% osmium tetroxide (OsO_4_). Samples were dehydrated through a graded ethanol series (20%, 40%, 60%, 80%, and 100%) for 15 min at each step, followed by two 15-min immersions in absolute ethanol. After drying, specimens were coated with platinum (Quorum Q150R ES; Quorum, East Sussex, UK) and examined by SEM (Hitachi SU8020; Hitachi, Tokyo, Japan).

### Combination with conventional antibiotics

The interaction between lipidated peptide and conventional antibiotics (cefoxitin, ciprofloxacin, and gentamicin) was assessed using a checkerboard microdilution method (Thitirungreangchai, Roytrakul & Aunpad, 2024). Briefly, bacterial suspensions (approximately 10^7^ CFU/mL) were inoculated into 96-well microtiter plates containing two-fold serial dilutions of antibiotic and peptide (1/128× MIC to 4× MIC). As previously described, MICs were determined after a 24-h incubation at 37 °C. All experiments were conducted in triplicate in three independent experiments. The interactions between peptides and antibiotics were quantified by calculating the fractional inhibitory concentration (FIC) index using the formula below (Thitirungreangchai, Roytrakul & Aunpad, 2024):

${\rm FIC\; index = FICA + FICB}$

FICA = (MIC of candidate peptide in combination/MIC of candidate peptide alone)

FICB = (MIC of antibiotic in combination/MIC of antibiotic alone)

FICI values were interpreted as follows: FICI ≤ 0.5 indicated synergistic; 0.5 < FICI ≤ 1 indicated additive; 1 < FICI ≤ 4 indicated indifferent; and FICI > 4 indicated antagonistic.

### Statistical analysis

All experiments were conducted in triplicate in three independent experiments, and data are presented as the mean ± standard deviation (SD). One-way ANOVA followed by Tukey’s *post hoc* test was used for statistical comparisons, performed using GraphPad Prism software (version 7.0; GraphPad Software, Boston, MA, USA). A statistically significant result was established as a *p-*value < 0.05.

## Results

### Lipidated peptides exhibited enhanced antibacterial activity against ESKAPE pathogens

The antibacterial efficacy of P7 and lipidated P7 peptides was evaluated against a panel of ESKAPE pathogens; melittin, a well-known broad-spectrum antimicrobial peptide (Guha et al., 2021) was used as a positive control. All lipidated derivatives exhibited markedly enhanced antibacterial activity relative to unmodified P7 peptide against five out of the six ESKAPE pathogens (*E. faecium*, *S. aureus*, *K. pneumoniae*, *A. baumannii*, and *E. cloacae*) with both MICs and MBCs within the range of 15.63 to 62.5 μg/mL (Table 2). For comparison, MIC and MBC values for melittin ranged from 7.81 to 125 μg/mL, indicating potent activity by the P7 derivatives against the panel of pathogens. Of the bacteria tested, *K. pneumoniae* (ATCC 27736), *S. aureus* (ATCC 25923), and *P. aeruginosa* (DMST 15501) showed comparable susceptibility, with similarly low MIC values. However, *K. pneumoniae* exhibited the greatest reduction in MIC relative to the unmodified P7 peptide and was therefore selected for further mechanistic and functional investigations.

**Table 2:** Minimum inhibitory concentrations (MICs) and minimum bactericidal concentrations (MBCs) of P7, lipidated P7 peptides, and melittin against ESKAPE pathogens.

Bacterial strain	MIC (MBC) in μg/mL				
	P7	C8-P7	C9-P7	C10-P7	Melittin
*Enterococcus faecium* ATCC 700221	62.5 (>250)	15.63 (31.25)	15.63 (31.25)	15.63 (31.25)	15.63 (15.63)
*Staphylococcus aureus* ATCC 25923	62.5 (62.5)	15.63 (15.63)	15.63 (15.63)	15.63 (15.63)	7.81 (7.81)
*Klebsiella pneumoniae* ATCC 27736	125 (250)	15.63 (15.63)	15.63 (15.63)	15.63 (31.25)	15.63 (15.63)
*Acinetobacter baumannii* DMST 43250	62.5 (62.5)	31.25 (31.25)	15.63 (15.63)	15.63 (15.63)	7.81 (7.81)
*Pseudomonas aeruginosa* DMST 15501	15.63 (15.63)	15.63 (15.63)	15.63 (15.63)	15.63 (31.25)	31.25 (31.25)
*Enterobacter cloacae* DMST 553	>250 (>250)	62.5 (62.5)	62.5 (62.5)	31.25 (31.25)	125 (125)

### Comparative assessment of antimicrobial potency following enzymatic degradation

The proteolytic stability of P7 and its lipidated derivatives (C8-P7, C9-P7, and C10-P7) was evaluated against *K. pneumoniae* ATCC 27736 following treatment with the digestive enzymes (trypsin and pepsin) (Table 3). Exposure to trypsin resulted in a complete loss of antibacterial activity for all peptides, with MIC and MBC values exceeding 250 μg/mL. Following pepsin treatment, a marked reduction in activity was also observed. The parent peptide P7 exhibited a complete loss of activity (the MIC increased from 125 μg/mL to >250 μg/mL), whereas the lipidated analogs (C8-P7, C9-P7, and C10-P7) retained minimal activity, with MIC value of 250 μg/mL. This corresponds to an approximately 16-fold reduction in potency compared to their original MIC values (15.63 μg/mL). Collectively, these results indicate that N-terminal lipidation does not confer substantial proteolytic stability to the P7 scaffold under gastric or intestinal enzyme conditions, as all variants exhibited near-complete loss of antibacterial activity following enzymatic exposure.

**Table 3:** Minimum inhibitory concentrations (MICs) and minimum bactericidal concentrations (MBCs) of P7 and lipidated P7 peptides after treatment with proteolytic enzymes against *K. pneumoniae* ATCC 27736.

*Klebsiella pneumoniae* ATCC 27736	MIC (MBC) in μg/mL			
	P7	C8-P7	C9-P7	C10-P7
Trypsin	>250 (>250)	>250 (>250)	>250 (>250)	>250 (>250)
Pepsin	>250 (>250)	250 (>250)	250 (250)	250 (250)

### Lipidated peptides exhibited α-helical amphipathic conformations in membrane-mimetic environments

The secondary structures of P7 and its lipidated analogs were analyzed by circular dichroism (CD) spectroscopy under three distinct conditions: 50% TFE, 30 mM SDS, and PBS, with melittin used as a positive control. As shown in Fig. 1, all three lipidated peptides displayed a characteristic negative peak around 195 nm in PBS, indicative of a random coil conformation in aqueous conditions. Conversely, spectra obtained in 50% TFE and 30 mM SDS revealed a marked shift, featuring a positive peak near 190 nm and two strong negative peaks at 208 and 222 nm, both hallmarks of α-helical structures. These results indicated that the lipidated peptides adopted amphipathic α-helical conformations under membrane-mimicking environments, consistent with the structural behavior of both the parent P7 peptide and melittin. This conformational flexibility likely facilitates membrane interaction and contributes to their antimicrobial activity.

**Figure 1:**
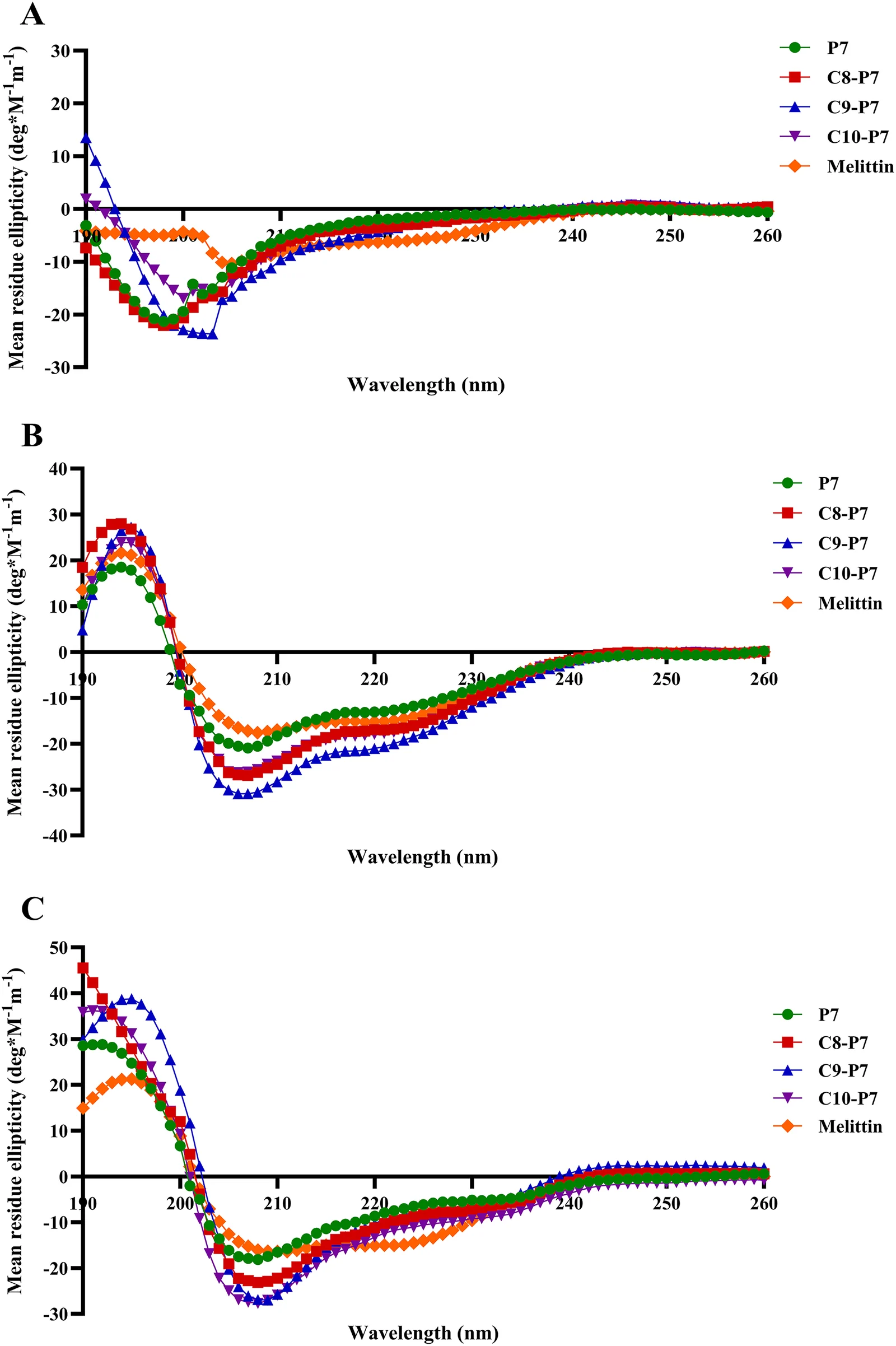
CD spectra of P7, lipidated P7 peptides, and melittin in PBS (A), 50% TFE (B), and 30 mM SDS (C). Spectra were recorded at 25 °C using a 0.1 cm quartz cuvette at 10 nm/min over 190–260 nm.

### C8-P7 peptide exhibited the lowest hemolytic activity among lipidated peptides

The hemolytic activities of P7, lipidated P7 peptides, and melittin were evaluated using hRBCs to evaluate their cytotoxic potential. Peptides were tested at varying concentrations from 0.98 to 250 μg/mL, with melittin included as a positive control. As illustrated in Fig. 2, at the MIC of 15.63 μg/mL, C8-P7 induced 19.58 ± 4.95% hemolysis, which was substantially lower than that induced by C9-P7 (31.31 ± 0.79%) and C10-P7 (40.43 ± 7.58%). In contrast, melittin caused complete hemolysis at the same concentration, whereas the parent P7 peptide induced 2.89 ± 0.95% hemolysis. At higher concentrations, C8-P7 induced complete hemolysis only at 250 μg/mL, whereas both C9-P7 and C10-P7 caused complete hemolysis at 125 μg/mL. Conversely, the parent P7 peptide continued to exhibit low hemolytic activity, causing only 12.76 ± 0.84% hemolysis at 250 μg/mL.

**Figure 2:**
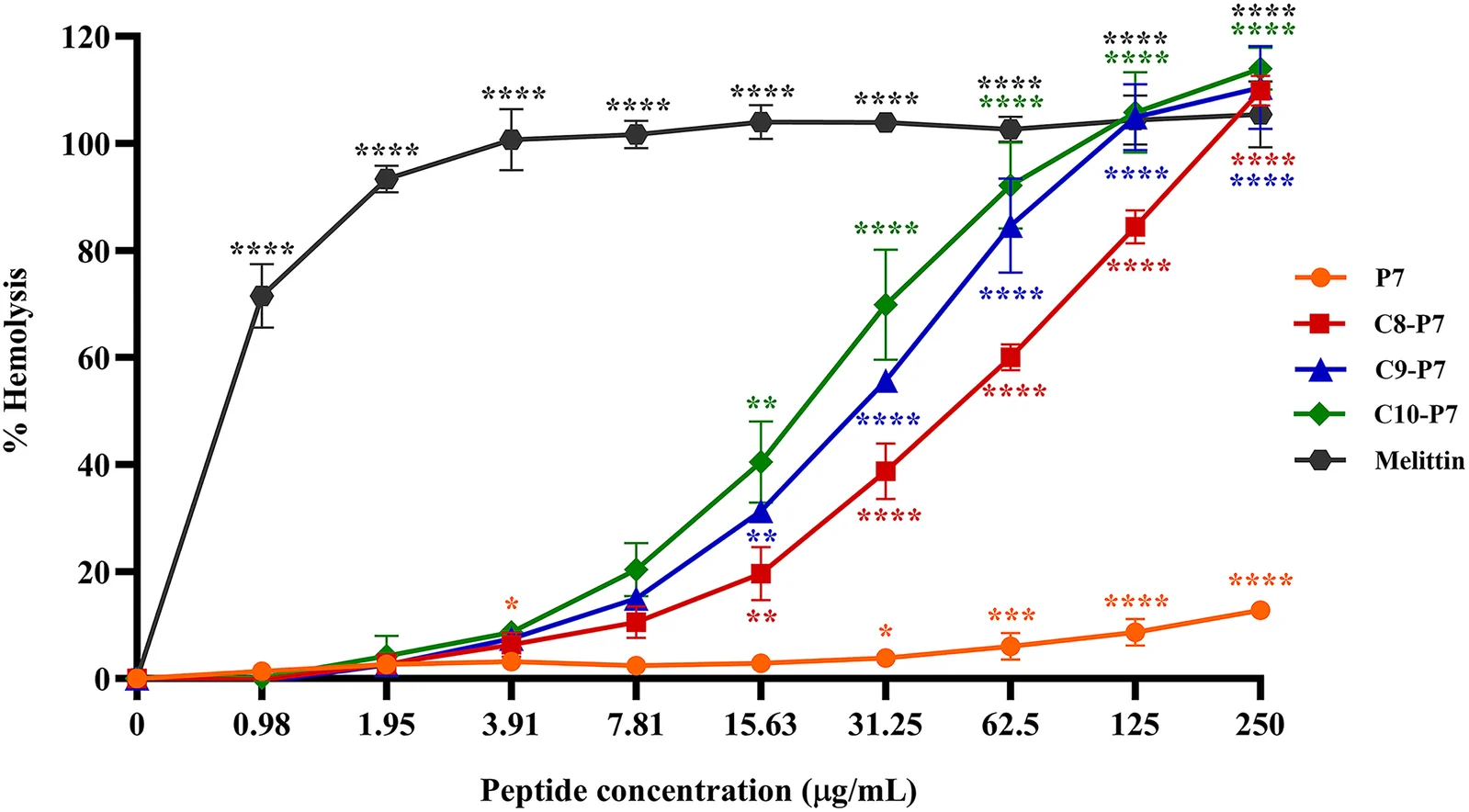
Hemolytic activity of P7, lipidated P7 peptides, and melittin against human red blood cells. Data are presented as mean ± SD from three independent biological replicates. Statistical significance compared to the negative control: *p < 0.05, **p < 0.01, ***p < 0.001, ****p < 0.0001.

The therapeutic index (TI) was calculated as a comparative measure of antibacterial selectivity. As summarized in Table 4, the lipidated derivatives overall exhibited low TI values (0.15–0.50), all lower than that of the parent peptide, indicating a limited safety margin. Among the variants, C8-P7 showed relatively higher TI values (0.30 against Gram-negative and 0.50 against Gram-positive bacteria) compared with C9-P7 and C10-P7 (0.15–0.25, and 0.18–0.25, respectively).

**Table 4:** Minimum hemolytic concentration (MHC), geometric mean MIC (GM), and therapeutic index (TI) of P7, lipidated P7 peptides, and melittin against ESKAPE pathogens.

	MIC in μg/mL				
	P7	C8-P7	C9-P7	C10-P7	Melittin
${\rm MHC}_{10}$^a^ (μg/mL)	250	7.81	3.91	3.91	<0.98
GM^b^ (Gr.− strains)	125.01	26.28	26.28	22.10	22.10
Therapeutic index (TI)^c^ (Gr.− strains)	2.00	0.30	0.15	0.18	0.02
GM^d^ (Gr.+ strains)	62.50	15.63	15.63	15.63	11.05
Therapeutic index (TI)^e^ (Gr.+ strains)	4.00	0.50	0.25	0.25	0.04

**Notes:**

a MHC was defined as the lowest concentration that resulted in 10% hemolysis of human red blood cells (hRBC). When 10% hemolysis was not observed at 250 μg/mL, a value of 500 μg/mL was used for therapeutic index calculation. Conversely, when 10% hemolysis occurred at a concentration below 0.98 μg/mL, a value of 0.49 μg/mL was used in the calculation.

b GM (Gr.− strains) denotes all Gram-negative strains’ geometric mean of MIC values. When no detectable MIC is observed at the maximum concentration of 250 μg/mL, a value of 500 μg/mL is used for therapeutic index calculation.

c Therapeutic index (Gr.− strains) is the ratio of the MHC to the geometric mean of MICs from Gram-negative strains.

d GM (Gr.+ strains) denotes all Gram-positive strains’ geometric mean of MIC values.

e Therapeutic index (Gr.+ strains) is the ratio of the MHC to the geometric mean of MICs from all Gram-positive strains.

### Lipidated peptides disrupted bacterial membrane integrity *via* permeabilization and depolarization

The membrane-disruptive activity of P7 and its lipidated derivatives was evaluated in *K. pneumoniae* by assessing membrane permeabilization and depolarization using PI and BOX fluorescent dyes, respectively. Untreated control cells maintained >98% viability throughout the 4-h observation period, confirming intact membrane integrity (Fig. 3). In contrast, treatment with 1× MIC of C8-P7, C9-P7, and C10-P7 each induced rapid and substantial increases in the combined PI and BOX fluorescence signal as early as 0.5 h (81.00 ± 0.30%, 87.23 ± 1.39%, and 87.37 ± 0.38%, respectively), indicating an early onset of membrane disruption. These responses were significantly higher than those observed for the unmodified P7 peptide (33.47 ± 1.40%). At 4 h post-treatment, these elevated fluorescence levels of the lipidated peptides persisted (96.40 ± 0.10%, 98.13 ± 2.19%, and 95.67 ± 0.51%, respectively), while P7 reached 86.90 ± 1.66% (similar to the melittin-treated positive control). These results demonstrated that lipidation markedly enhanced the membrane-disruptive property of P7 peptides, suggesting that their increased bactericidal activity was through accelerated and sustained membrane permeabilization and depolarization.

**Figure 3:**
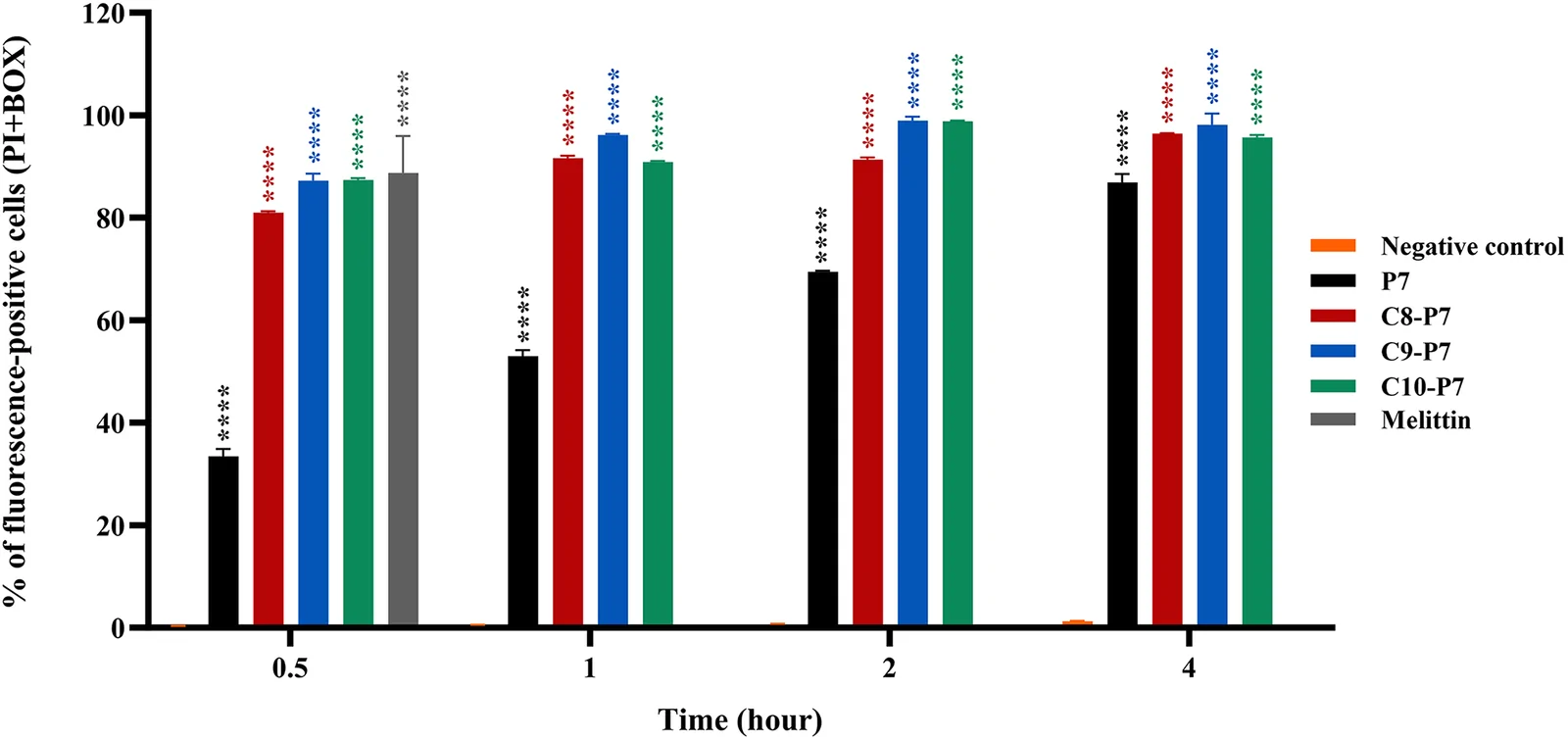
Flow cytometric analysis of *K. pneumoniae* ATCC 27736 treated with P7, lipidated P7 peptides, or melittin at 1× MIC. The combined PI and BOX fluorescence signals were assessed at 0.5, 1, 2, and 4 h. Data represent mean ± SD from three independent biological replicates. Statistical significance compared to the negative control: ****p < 0.0001.

### Cytotoxicity and safety evaluation of C8-P7

The cytotoxicity and safety profile of C8-P7 were evaluated using the MTT assay in L929 mouse fibroblast cells, with melittin serving as a positive toxic control. Following 24 h of exposure, cell viability was measured to establish a comprehensive safety profile. To further characterize the host cell interactions, a full dose-response curve was generated (Fig. 4). The half-maximal inhibitory concentration (IC_50_) for C8-P7 was calculated to be 36.44 μg/mL, whereas the positive toxic control, melittin, exhibited a calculated IC_50_ of 16.23 μg/mL under identical conditions. While C8-P7 demonstrated lower cytotoxicity than melittin, these results indicate that the concentration required for pathogen eradication is close to the threshold of host cell cytotoxicity. These findings indicate a narrow therapeutic window for this variant, reflecting the concurrent increase in both antimicrobial potency and mammalian cell toxicity following lipidation.

**Figure 4:**
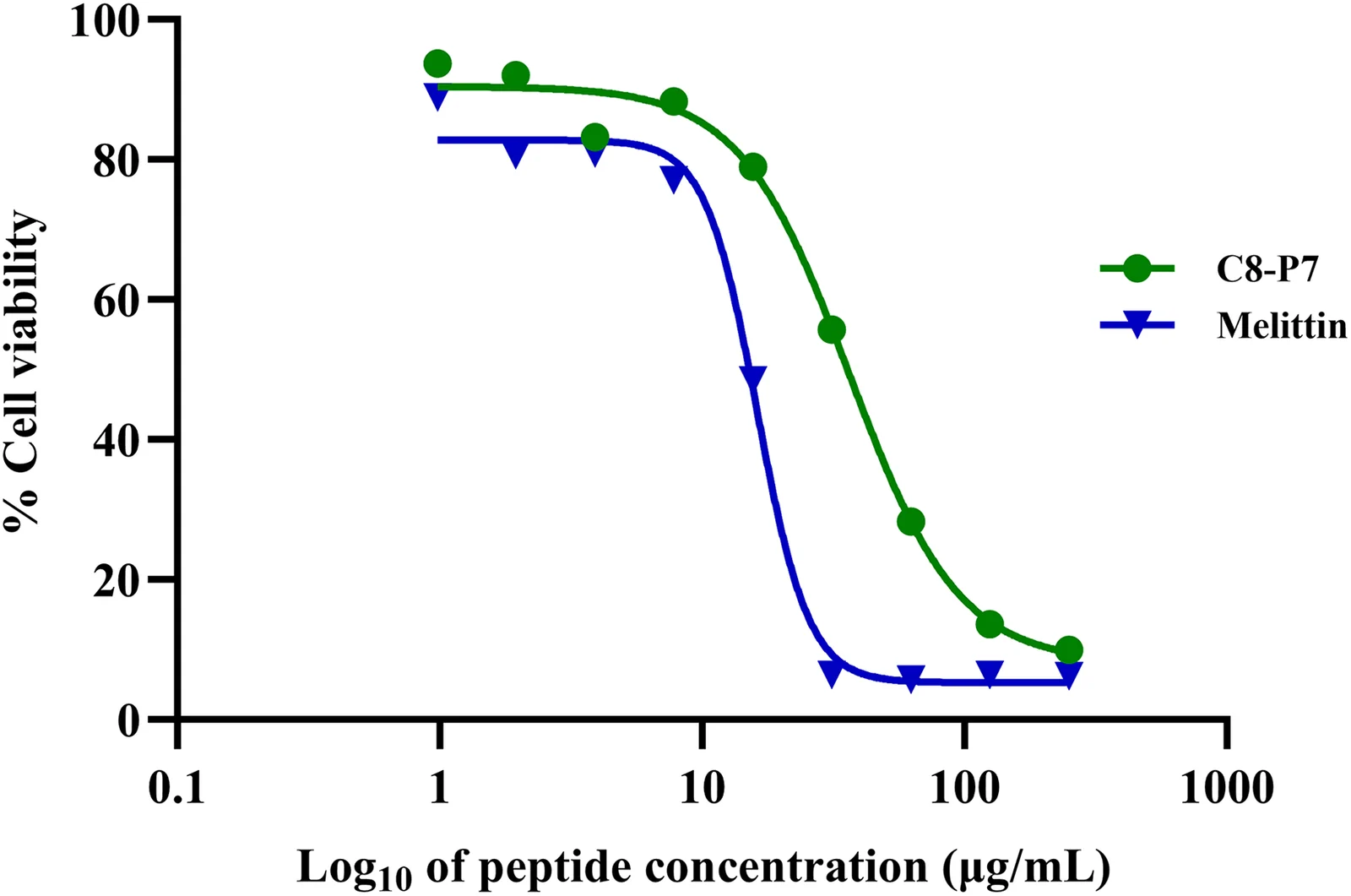
Dose-response curves of C8-P7 and melittin against L929 mouse fibroblast cells. Data represent the mean from three independent biological replicates.

### Bactericidal kinetics of the C8-P7 peptide against *K. pneumoniae*

Time-dependent killing activity of the C8-P7 peptide against *K. pneumoniae* was assessed by monitoring bacterial viability following exposure to 1× MIC at multiple time points (0, 0.5, 1, 2, 4, 6, and 24 h). As presented in Fig. 5, the untreated control cultures exhibited a steady increase in viable cell counts over time, confirming active bacterial proliferation. In contrast, treatment with C8-P7 peptide led to a progressive decline in viable bacterial cell counts. Notably, no viable bacteria were detected after 24 h of peptide exposure. These results demonstrated that C8-P7 peptide exerted a potent, time-dependent bactericidal effect against *K. pneumoniae*, effectively eliminating the bacterial population within 24 h.

**Figure 5:**
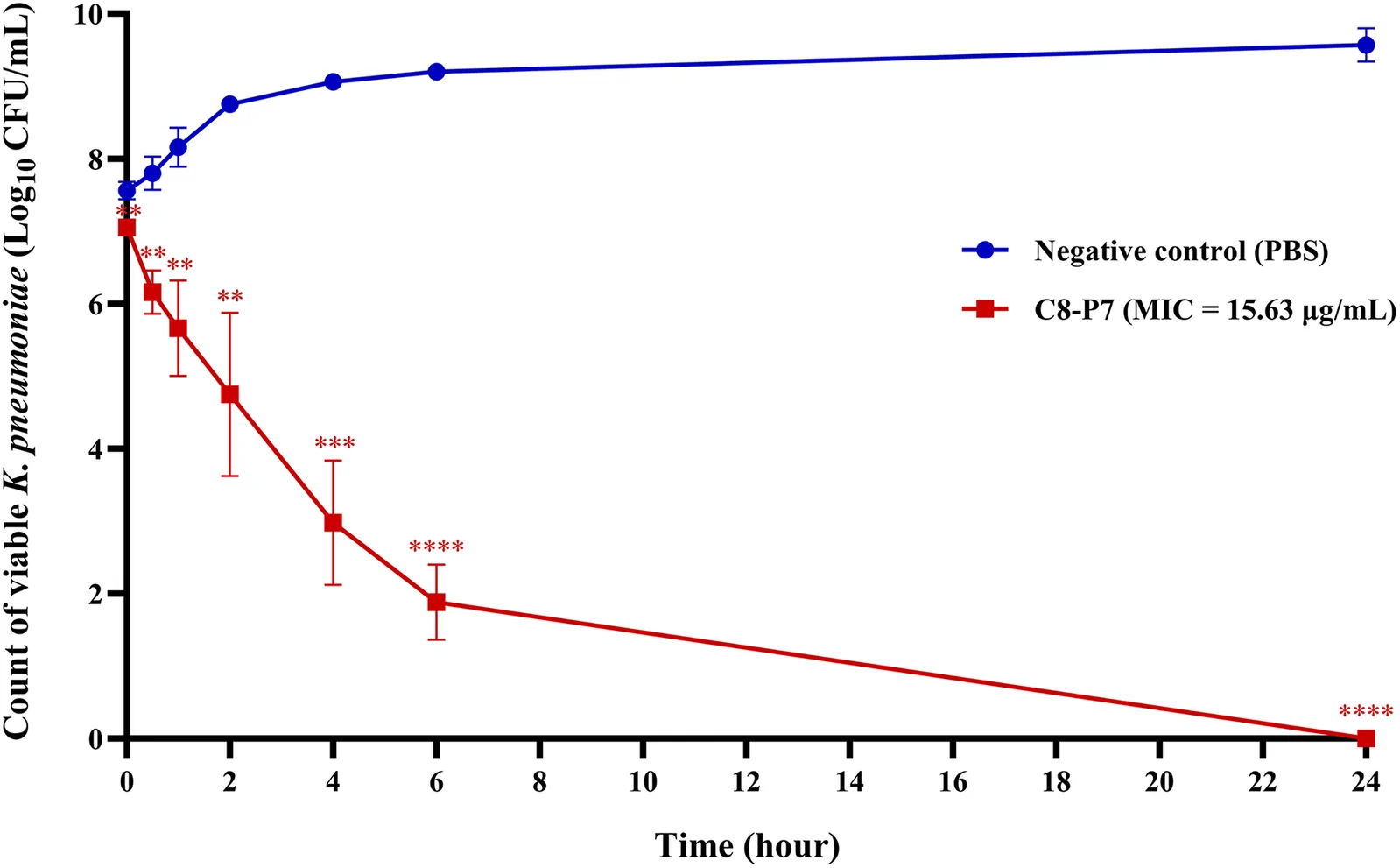
Time-kill kinetics of the C8-P7 peptide at 1× MIC (and negative control) against *K. pneumoniae* ATCC 27736. Bacterial viability was evaluated at 0, 0.5, 1, 2, 4, 6, and 24 h post-treatment. Data are presented as mean ± SD from three independent biological replicates. Statistical significance compared to the negative control: **p < 0.01, ***p < 0.001, ****p < 0.0001.

### Structural alterations in *K. pneumoniae* induced by the C8-P7 peptide

Ultrastructural alterations and membrane integrity of *K. pneumoniae* were examined by transmission electron microscopy after 2 h of treatment with the C8-P7 peptide at 1× MIC. Untreated cells exhibited well-preserved morphology (Figs. 6A and 6B), characterized by intact cell membranes and uniformly distributed intracellular contents. In contrast, bacterial cells treated with the C8-P7 peptide displayed marked structural disruptions (Figs. 6C–6F), including compromised membrane integrity, thinning of the cell envelope, irregular membrane disruptions with visible pore formation, leakage of cytoplasmic contents, and the presence of electron-lucent zones containing inclusion bodies. These findings of membrane damage and intracellular disorganization support the suggested bactericidal mechanism of action of C8-P7 peptide.

**Figure 6:**
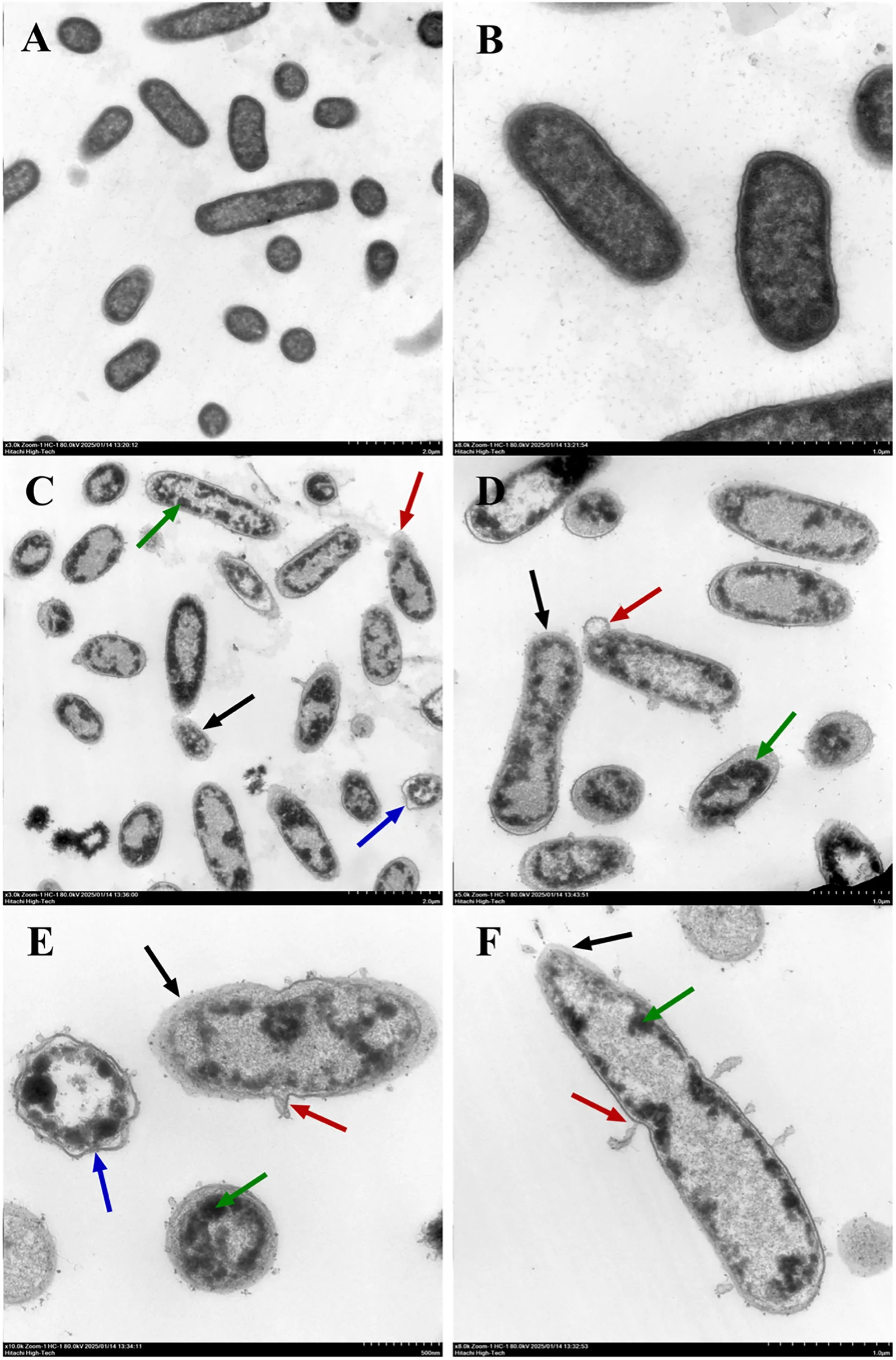
TEM images of *K. pneumoniae* ATCC 27736. (A and B) Untreated cells. (C–F) Cells treated with 1× MIC of C8-P7 for 2 h. Red, blue, green, and black arrows indicate intracellular leakage, membrane deformation, inclusion bodies, and membrane thinning, respectively.

Morphological alterations in *K. pneumoniae* following C8-P7 peptide treatment were further examined using scanning electron microscopy, under the same experimental conditions as the TEM analysis. As shown in Fig. 7A, untreated cells displayed characteristic short, rod-shaped morphology with smooth and intact cell surfaces. In contrast, C8-P7-treated cells exhibited pronounced structural damage (Fig. 7B), including membrane roughening, surface distortion, and clear signs of membrane disruption. These changes were accompanied by visible leakage of intracellular contents and evidence of cellular lysis, supporting the conclusion that C8-P7 exerted its bactericidal effect through direct disruption of bacterial membrane integrity.

**Figure 7:**
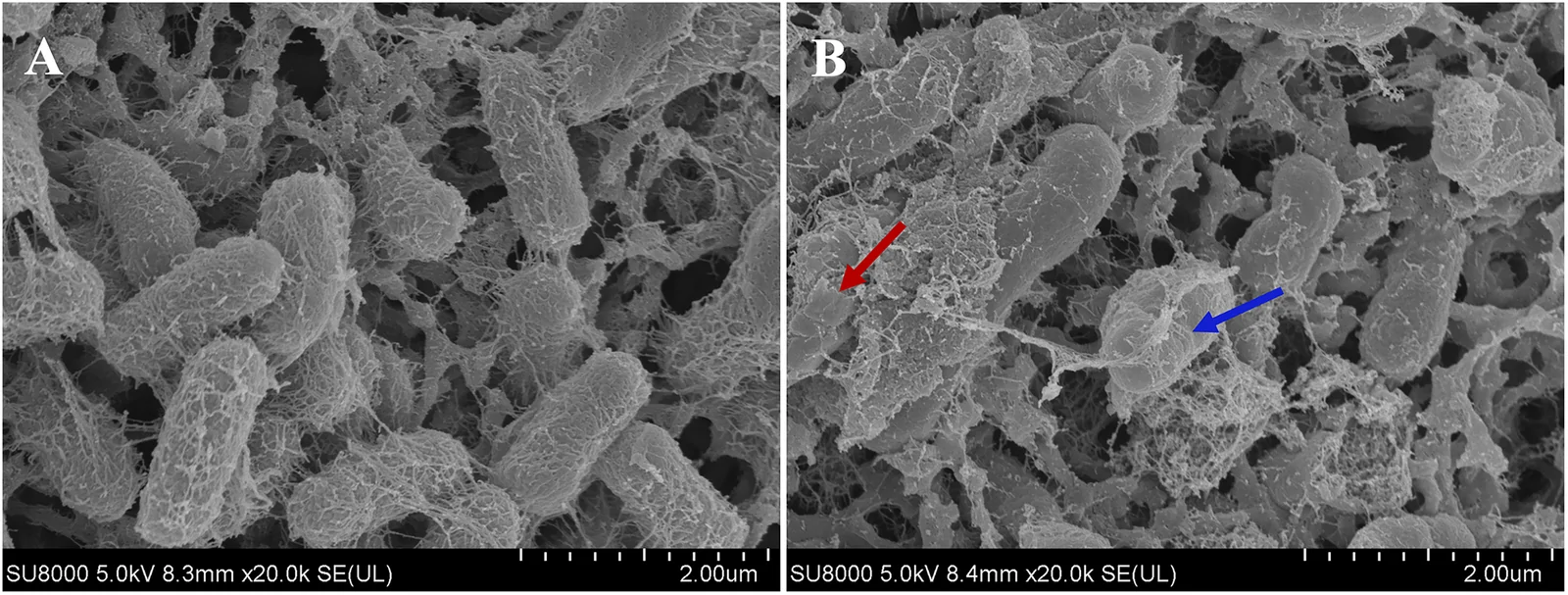
SEM images of *K. pneumoniae* ATCC 27736. (A) Untreated cells. (B) Cells treated with 1× MIC of C8-P7 for 2 h. Red and blue arrows indicate lysed cells and membrane distortion, respectively.

### Additive antibacterial activity of C8-P7 peptide in combination with ciprofloxacin and gentamicin against *K. pneumoniae*

Synergistic potential of the C8-P7 peptide, in combination with clinically relevant antibiotics (cefoxitin, ciprofloxacin, and gentamicin) against *K. pneumoniae* was assessed using a checkerboard microdilution method. The fractional inhibitory concentration index (FICI) quantified the combined effect with each antimicrobial agent (summarized in Table 5). The C8-P7 peptide, when combined with ciprofloxacin or gentamicin, exhibited an additive effect, resulting in FICI values in the range of 0.51 to 0.75. In contrast, the combination of C8-P7 peptide and cefoxitin resulted in an indifferent interaction (FICI value of 1.02). These findings suggested that C8-P7 may enhance the antibacterial efficacy of ciprofloxacin and gentamicin, providing the basis for a potential combination strategy for treating infections caused by *K. pneumoniae*.

**Table 5:** *In vitro* interactions between C8-P7 and three conventional antibiotics against *K. pneumoniae* ATCC 27736.

Antibiotic	MIC of the antibiotic (μg/mL)	MIC of the antibiotic in combination with C8-P7 (μg/mL)	MIC of the C8-P7 in combination with antibiotics (μg/mL)	FICI	Effect
Gentamicin	0.98	0.49	3.91	0.75	Additive
Ciprofloxacin	0.98	0.015	7.81	0.51	Additive
Cefoxitin	3.91	0.06	15.63	1.02	Indifferent

## Discussion

The WHO has classified ESKAPE pathogens as “priority status” threats due to their increasing multidrug resistance (De Oliveira et al., 2020). This underscores the urgent need to develop effective alternatives to current antibiotics. Antimicrobial peptides are attractive therapeutic candidates owing to their broad-spectrum efficacy and low risk of resistance development (Moretta et al., 2021). However, despite their considerable therapeutic potential, clinical application of AMPs is limited by relatively low antibacterial activity and poor stability (particularly due to their susceptibility to proteolytic degradation) (Rima et al., 2021). Lipidation, which involves conjugating a hydrophobic fatty acid to a peptide’s N- or C-terminus, is among the most promising strategies to overcome these limitations. This modification is designed to enhance membrane interactions, increases antibacterial potency, and potentially improve resistance to enzymatic degradation. For instance, Liu et al. (2020) observed that conjugating a fatty acid to the N-terminus of antimicrobial peptides M3.6, M4.7, and M5.7 enhanced both their antibacterial activity and proteolytic stability. Compared to their native counterparts, those lipidated peptides exhibited increased resistance to degradation by trypsin and chymotrypsin (Liu et al., 2020). In a related study, Dewangan et al. (2022) conjugated fatty acids of varying chain lengths to the N-terminus of PR-Spn, which enhanced its antibacterial efficacy against both Gram-positive and -negative bacteria. Those lipidated derivatives also demonstrated a resistance to trypsin-mediated proteolysis. These findings support lipidation as a strategic approach to concurrently improve the antimicrobial efficacy and proteolytic stability of various AMP scaffolds (Rounds & Straus, 2020; Selvaraj & Chen, 2023).

Our group previously reported that P7, a cathelicidin-derived peptide, exhibited strong antibacterial activity against drug-resistant *Salmonella enterica* serovar Typhimurium, with minimal hemolytic effects (Klubthawee & Aunpad, 2020). However, its efficacy against ESKAPE pathogens remained uncharacterized. These pathogens represent a significant clinical challenge due to their advanced multi-drug resistance (MDR) mechanisms. To address this challenge, the current study assessed P7’s activity against these pathogens and evaluated whether N-terminal fatty acid conjugation could enhance its antimicrobial potency and proteolytic stability. The parent P7 peptide was conjugated with fatty acids containing 8, 9, or 10 carbon atoms, resulting in significantly enhanced antibacterial efficacy and an activity spectrum that included five of the six ESKAPE pathogens. However, *P. aeruginosa* remained unaffected by lipidation, exhibiting MIC values comparable to those of the unmodified P7. This limited susceptibility was likely attributable to the bacterium’s intrinsically low outer membrane permeability, estimated to be only about 8% of that of *Escherichia coli*, which severely restricts uptake of many antimicrobial agents. This functional barrier of *P. aeruginosa* is further reinforced by the absence of large, nonspecific porins, such as OmpF and OmpC, which are present in *E. coli* and facilitate passive diffusion of small hydrophilic molecules (Chevalier et al., 2017). Our findings regarding *P. aeruginosa* are consistent with previous reports on lipidated AMPs, including those modified with medium-chain fatty acids. For instance, Yang et al. (2023a) showed that N-terminal conjugation of lauric acid to the CL5 peptide enhances overall activity against Gram-negative bacteria; however the magnitude of improvement was strain-dependent, with only limited or no substantial enhancement observed against *P. aeruginosa* compared to the parent peptide. Similarly, Liao et al. (2025) reported that C8 and C10 derivatives of the B1 peptide exhibited increased activity against *S. aureus* and *K. pneumoniae*, yet failed to improve efficacy against *A. baumannii* and *P. aeruginosa*. Taken together, these findings indicate that the effects of medium-chain fatty acid conjugation are not uniformly conserved across ESKAPE pathogens but are instead highly context-dependent. While lipidation can substantially enhance activity against certain Gram-positive and Gram-negative bacteria, its impact against *P. aeruginosa* appears consistently limited across multiple peptide scaffolds. This trend likely reflects the distinctive outer membrane architecture of *P. aeruginosa*, characterized by a densely packed lipopolysaccharide layer and adaptive resistance mechanisms, which collectively restrict peptide access and diminish the efficacy of lipidation-based optimization strategies.

Among the pathogens tested, the reduction in MIC values following lipidation was most pronounced against *K. pneumoniae*, with up to an 8-fold decrease compared to the parent P7 peptide. The clinical threat of *K. pneumoniae* is escalating, driven by its resistance to last-resort antibiotics and its acquisition in both community and healthcare settings. Its rapid dissemination and resistance to critical antimicrobials make it a key target for novel therapeutic strategies (Chatupheeraphat et al., 2023). The observed enhancement in antibacterial activity by AMPs aligns with previous reports indicating that their lipidation improves efficacy by increasing hydrophobic interactions with bacterial cell membranes, thereby facilitating membrane insertion and disruption (Grimsey et al., 2020; Rounds & Straus, 2020). For instance, when Ma et al. (2022) modified the N-terminus of the antimicrobial peptide CAMEL by conjugating fatty acids ranging from C4 to C16, they found that the C4–C12 lipidated analogs exhibited enhanced antimicrobial activity against *E. coli*. Similarly, Zhang et al. (2023) evaluated the antibacterial activity of lin-n-PE2 (where n is the number of carbon atoms in the fatty acyl chain) compared with lin-PE2 alone and showed that analogues with *n* = 8, 9, or 10 had improved activity against both Gram-positive and -negative bacteria. In another study, Zhong et al. (2019) conjugated fatty acids to the N-terminus of anoplin-D4, 7. Peptides modified with shorter fatty acid chains (C4 or C6) exhibited weak antimicrobial activity, while those with longer chains (C8–C12) showed progressively stronger antibacterial effects.

Although lipidation has been widely reported to enhance proteolytic stability in certain antimicrobial peptides (Cai et al., 2025; Dewangan et al., 2022; Liu et al., 2020; Selvaraj & Chen, 2023), the effect is not universal. In the present study, N-terminal lipidation did not improve the stability of the P7 scaffold, as all variants exhibited substantial loss of antibacterial activity following enzymatic treatment. Notably, the lipidated derivatives showed a greater relative reduction in potency, with MIC values increasing approximately 16-fold, whereas the parent peptide displayed only an approximate two-fold increase. These findings suggest that lipidation alone is insufficient to protect the peptide from enzymatic degradation, and may not effectively prevent cleavage at susceptible sites within the sequence. Despite this, the lipidated analogs retained higher residual antibacterial activity than the parent peptide following pepsin exposure, owing to their significantly greater baseline potency. This indicates that, while lipidation does not necessarily confer intrinsic proteolytic stability, it may still provide a functional advantage by enhancing initial activity levels. Collectively, these results highlight that the impact of fatty acid conjugation is highly context-dependent and must be optimized in conjunction with peptide sequence and structural features to achieve an effective balance between stability and antimicrobial potency.

Most AMPs are unstructured in an aqueous environment but undergo conformational transitions, typically to α-helical structures, upon interacting with membrane-mimetic conditions. This structural shift facilitates membrane insertion and disruption, key steps in their antimicrobial mechanisms (Lyu et al., 2016; Ma et al., 2024). Fatty acid conjugation has been shown to further stabilize these α-helical conformations in lipid environments, thereby enhancing membrane affinity and activity (Reinhardt & Neundorf, 2016). In the present study, circular dichroism analysis revealed that all three lipidated P7 peptides (C8-, C9-, and C10-P7) retained random-coil structures in aqueous solution, similar to the parent P7 peptide. But they then adopted α-helical conformations in membrane-mimicking conditions (TFE and SDS), with helicity enhanced compared to that of the unmodified P7 peptide. The ability to form α-helices in membrane-like environments is widely regarded as critical for the lytic activity of AMPs, as this conformation promotes membrane disruption (Ma et al., 2024; Yang et al., 2023a). For instance, Yang et al. (2023a) demonstrated that the C12-conjugated CL5 peptide showed an enhanced helical content and increased membrane-disruptive capacity compared to its unconjugated parent.

The flow cytometry results demonstrate that lipidation significantly enhanced the membrane-disruptive activity of the P7 peptide against *K. pneumoniae*, as evidenced by rapid and sustained membrane permeabilization and depolarization. Using PI and BOX fluorescence assays, lipidated derivatives (C8-P7, C9-P7, and C10-P7) induced a substantial increase in fluorescence within 30 min of treatment, indicating early and effective compromise of bacterial membrane integrity. In contrast, the parent P7 peptide exhibited delayed and comparatively lower levels of membrane disruption, only approaching similar levels of membrane perturbation after 4 h of exposure. These findings suggest that the conjugation of medium-chain fatty acids to the N-terminus of P7 accelerated its interaction with and disruption of bacterial membranes. This enhancement was likely due to the increased hydrophobicity and amphipathic character imparted by the lipid tails, which facilitated deeper insertion into the lipid bilayer and destabilization of membrane architecture (Lin et al., 2023).

Lipidation of the P7 peptides led to a clear increase in hemolytic activity compared to the unmodified parent peptide. While C8-P7 exhibited the lowest hemolytic effect among the modified analogs, the observed hemolysis rate of approximately 20% at its MIC remains non-negligible and represents a potential limitation of this lead compound. A positive correlation was observed between fatty acid chain length and red blood cell lysis, likely because the increased hydrophobicity calculated to optimize bacterial membrane penetration simultaneously diminishes the peptide’s ability to preferentially target microbial bilayers over mammalian cell membranes. This trend aligns with previous studies demonstrating that longer acyl chains enhance membrane disruption across various cell types (Liu et al., 2020; Rounds & Straus, 2020). For instance, Liu et al. (2021) reported that conjugation with linoleic acid (C18) significantly increased the toxicity of Lfcin4 and Lfcin5 compared to their parent sequences. Similarly, Liao et al. (2025) observed that while a C8-conjugated B1 peptide maintained an MHC comparable to its parent, the C10 conjugate showed a markedly lower MHC signifying a higher hemolytic potency. To further characterize the safety profile, the cytotoxicity of the optimal variant, C8-P7, was evaluated against L929 mouse fibroblast cells. While C8-P7 demonstrated lower cytotoxicity than the melittin control, these findings indicate that the concentration required for pathogen eradication is close to the threshold of host cell cytotoxicity. This suggests a narrow therapeutic window for this variant, reflecting the concurrent increase in both antimicrobial potency and mammalian cell toxicity following lipidation. Consequently, the impact of lipidation is a balance between chain length and the specific peptide scaffold. Among the lipidated derivatives, C8-P7 emerged as the most balanced variant, providing sufficient hydrophobicity to penetrate the complex outer membranes of ESKAPE pathogens without reaching the excessive lipophilicity seen in C9-P7 and C10-P7. Collectively, these results underscore the dual-edged nature of lipidation; while essential for overcoming robust membrane barriers, the resulting shift in the hydrophobic-hydrophilic balance can narrow the safety margin. Although C8-P7 requires further structural refinement to improve selectivity and decouple antimicrobial potency from host cell toxicity, it serves as a promising scaffold that maintains a lower cytotoxic footprint than classical hemolytic peptides.

The time-kill kinetics experiment revealed that C8-P7 exerted a gradual, time-dependent bactericidal effect against *K. pneumoniae*, resulting in complete bacterial eradication within 24 h. This fast and sustained killing activity suggests a potent mode of action, primarily targeting the bacterial envelope. Such rapid bactericidal effects are characteristic of AMPs that disrupt bacterial membranes. For instance, chionodracine-derived peptides have demonstrated time- and concentration-dependent activity against *K. pneumoniae*, with higher concentrations resulting in faster killing kinetics (Olivieri et al., 2018). Similarly, the AMP Hs02 exhibits rapid bactericidal efficacy against carbapenem-resistant *K. pneumoniae*, achieving significant bacterial reduction within hours (Zhao et al., 2025). These findings support the notion that C8-P7’s bactericidal efficacy is mediated through membrane-targeting mechanisms. TEM and SEM also provided direct visual evidence supporting this hypothesis. Upon treatment with C8-P7, TEM analysis showed that *K. pneumoniae* cells had significant ultrastructural damage, including membrane deformation, the formation of small pores, and leakage of cytoplasmic contents, indicative of membrane effects which led to cell lysis. SEM analysis revealed further morphological abnormalities such as increased membrane roughness, membrane disruption, and cell membrane distortion. These TEM and SEM findings were supported by flow cytometry analyses using PI and BOX dyes, which detected membrane permeabilization and depolarization, respectively. C8-P7-treated cells showed a marked increase in uptake of both PI and BOX within the first 30 min of exposure, indicating early and substantial membrane disruption.

Prior research has demonstrated that the combination of AMPs with conventional antibiotics can enhance the latter’s antibacterial effects, decrease the required antibiotic dosage, and potentially delay the emergence of resistance (Duong, Gross & Siryaporn, 2021; Zhong et al., 2019). In this study, the C8-P7 peptide was evaluated in combination with cefoxitin, ciprofloxacin, and gentamicin, antibiotics commonly used to treat *K. pneumoniae* infections. Given the increasing prevalence of infections due to multidrug-resistant *K. pneumoniae*, such combinations are of growing clinical interest (Cireșă et al., 2024; Yang et al., 2023b). C8-P7 demonstrated additive effects when combined with ciprofloxacin and gentamicin, suggesting a complementary mode of action. These findings align with previous studies, such as Liu et al. (2020), who observed additive or synergistic interactions between lipidated peptides and antibiotics including rifampin, kanamycin, and polymyxin B. Lipidation increases affinity for membranes and disrupts their integrity, processes which may facilitate access to intracellular targets and increased intracellular antibiotic accumulation (Rounds & Straus, 2020). Such membrane-perturbing effects likely underline the improved efficacy of combination treatments, supporting the concept of lipidated AMPs used as adjuvants to conventional therapies (Zhong et al., 2019).

## Conclusions

This study demonstrated that N-terminal lipidation of the P7 peptide with medium-chain fatty acids (C8–C10) significantly enhances antimicrobial activity, particularly against *K. pneumoniae*. Among the modified variants, C8-P7 emerged as the most balanced candidate, exhibiting potent bactericidal effects primarily mediated through membrane disruption. However, the increase in cytotoxicity associated with longer acyl chains underscores a critical trade-off: while increased hydrophobicity is essential for overcoming robust bacterial barriers, it concurrently diminishes selectivity toward microbial membranes. Future investigations should prioritize refining the structure-activity relationships of these lipidated peptides to decouple antimicrobial potency from host cell toxicity, thereby expanding the currently narrow therapeutic index. Furthermore, evaluating *in vivo* efficacy and safety, alongside exploring synergistic applications with conventional antibiotics, will be essential steps in advancing these lipidated AMPs toward their potential as next-generation antimicrobial agents for clinical translation.

## Supplemental Information

10.7717/peerj.21717/supp-1 Supplemental Information 1 Supplementary data.

10.7717/peerj.21717/supp-2 Supplemental Information 2 Raw data of all experiments.

## Additional Information and Declarations

### Data Availability

The following information was supplied regarding data availability:

The data are available in the Supplemental Files.

The flow cytometry data is available at Figshare:

- Thammathaporn, Warintorn (2026). Lipid-tail modification of the antimicrobial peptide P7 enhances membrane disruption and antibacterial activity against *Klebsiella pneumoniae*. figshare. Dataset. https://doi.org/10.6084/m9.figshare.33181133.v1.
