# Supplementary material for: Lipid-tail modification of the antimicrobial peptide P7 enhances membrane disruption and antibacterial activity against Klebsiella pneumoniae
Source: PeerJ. 2026 Oct 7;14:e21717. doi: 10.7717/peerj.21717 (PMC13653536; doi:10.7717/peerj.21717)
Supplement: Supplementary file 1 [file peerj-14-21717-s001.pdf]

(A)

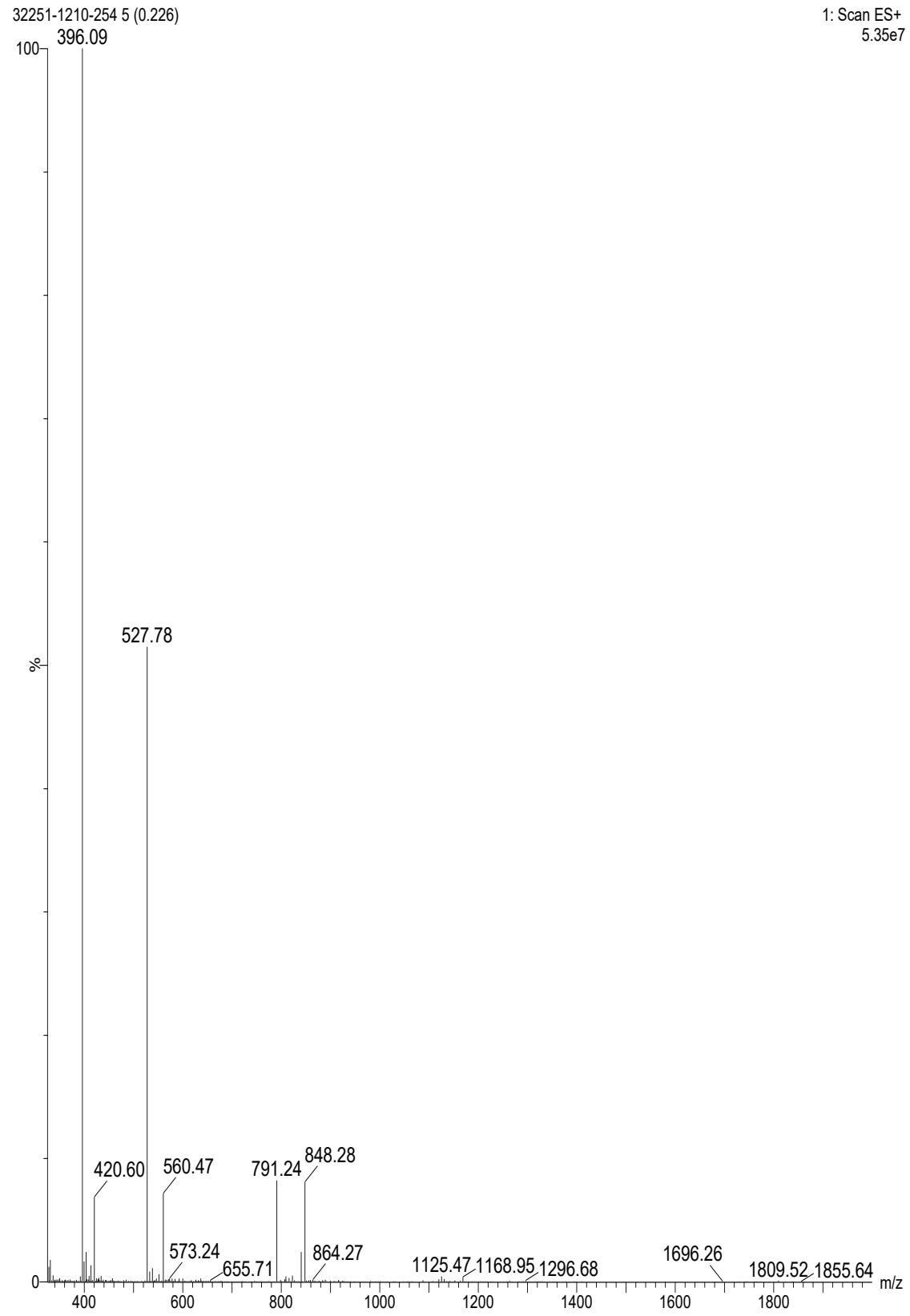

(B)

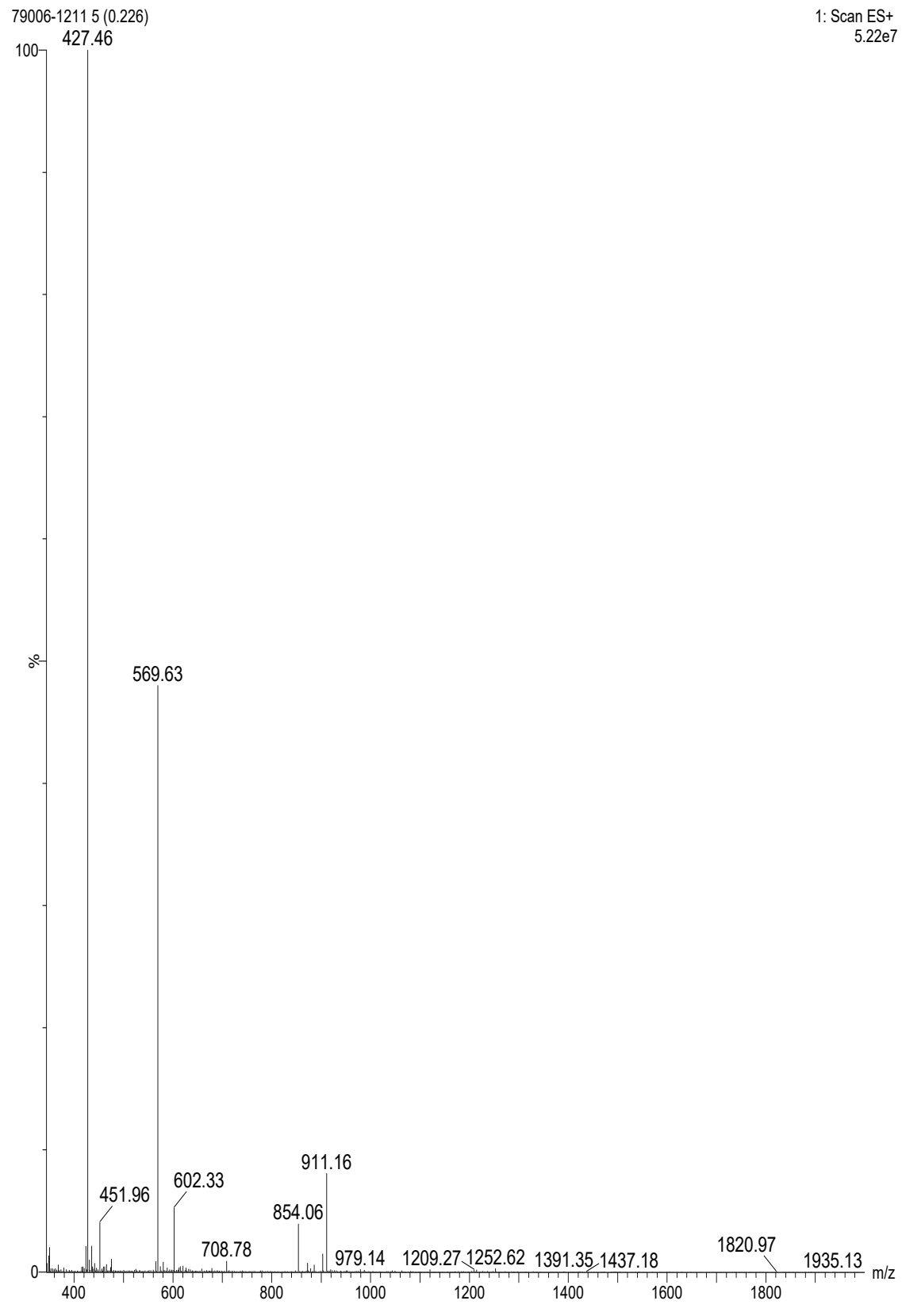

Fig. S1. ESI-MS of the peptides. (A) P7; (B) C8-P7; (C) C9-P7; and (D) C10-P7. (cont.)

(C)

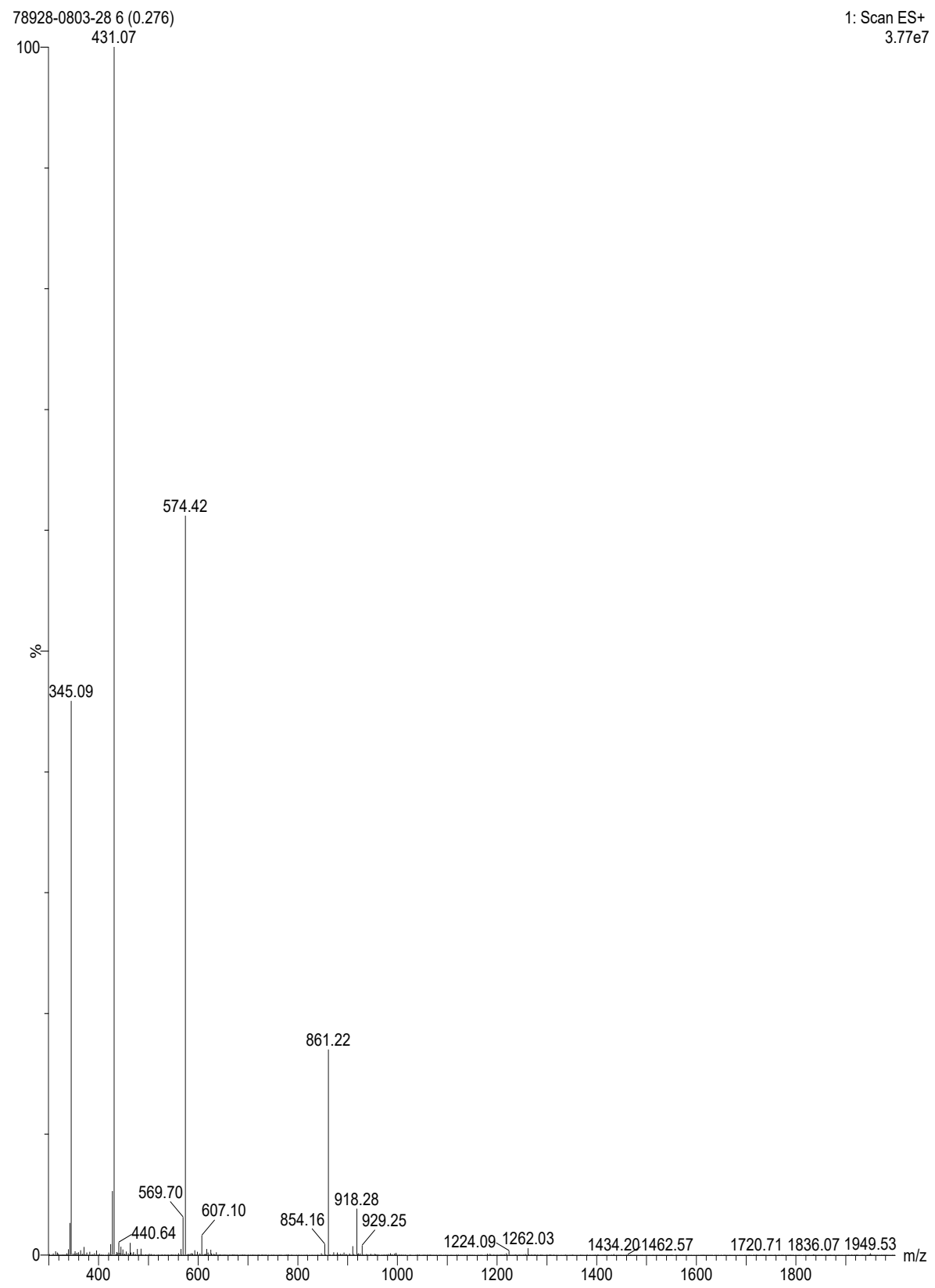

(D)

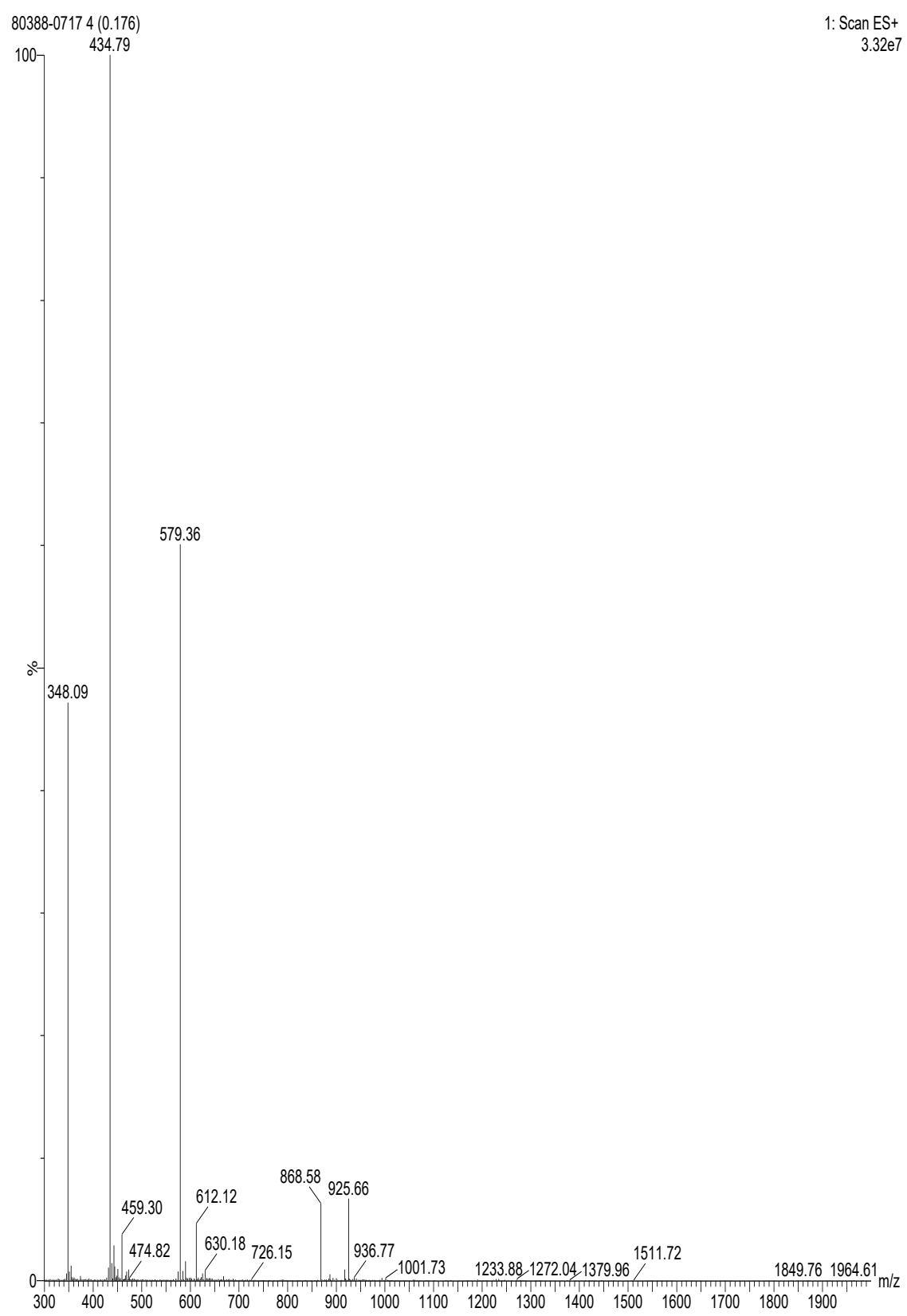

Fig. S1. ESI-MS of the peptides. (A) P7; (B) C8-P7; (C) C9-P7; and (D) C10-P7.

(A)

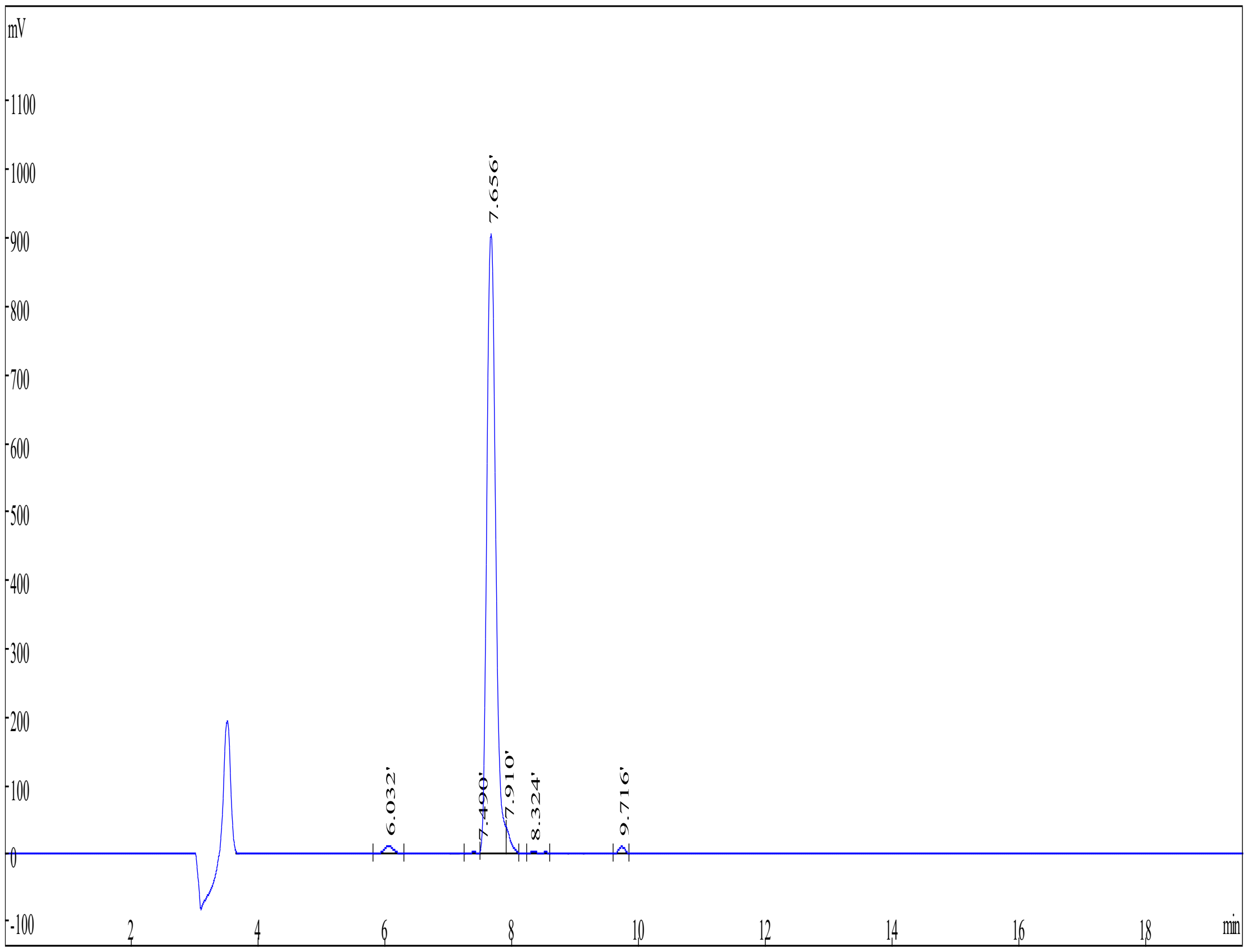

Fig. S2. (A) HPLC chromatogram of P7. HPLC analysis was performed using a Kromasil 100-5C18 column (4.6 mm × 250 mm, 5 µm) at 25 °C with detection at 220 nm, a flow rate of 1.0 mL/min, and an injection volume of 10 µL. Mobile phase A consisted of 0.1% TFA in acetonitrile, while mobile phase B consisted of 0.1% TFA in water. Linear gradient: 22% A/78% B at 0.0 min, 47% A/53% B at 20.0 min, and 100% A/0% B at 20.1 min; total run time 20 min.

(B)

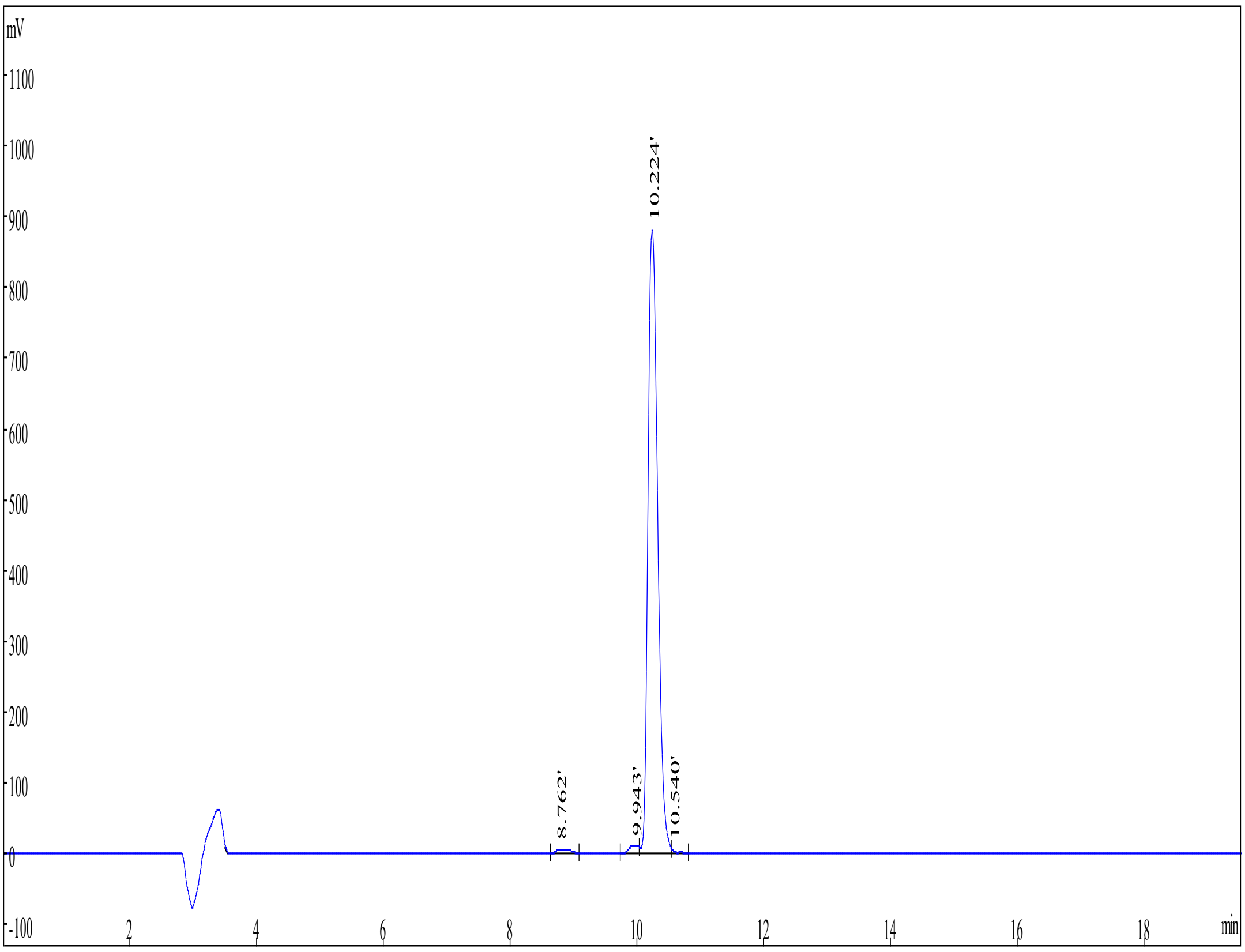

Fig. S2. (B) HPLC chromatogram of C8-P7. HPLC analysis was performed using a Kromasil 100-5C18 column (4.6 mm × 250 mm, 5 µm) at 25 °C with detection at 220 nm, a flow rate of 1.0 mL/min, and an injection volume of 10 µL. Mobile phase A consisted of 0.1% TFA in acetonitrile, while mobile phase B consisted of 0.1% TFA in water. Linear gradient: 35% A/65% B at 0.0 min, 60% A/40% B at 20.0 min, and 100% A/0% B at 20.1 min; total run time 20 min.

(C)

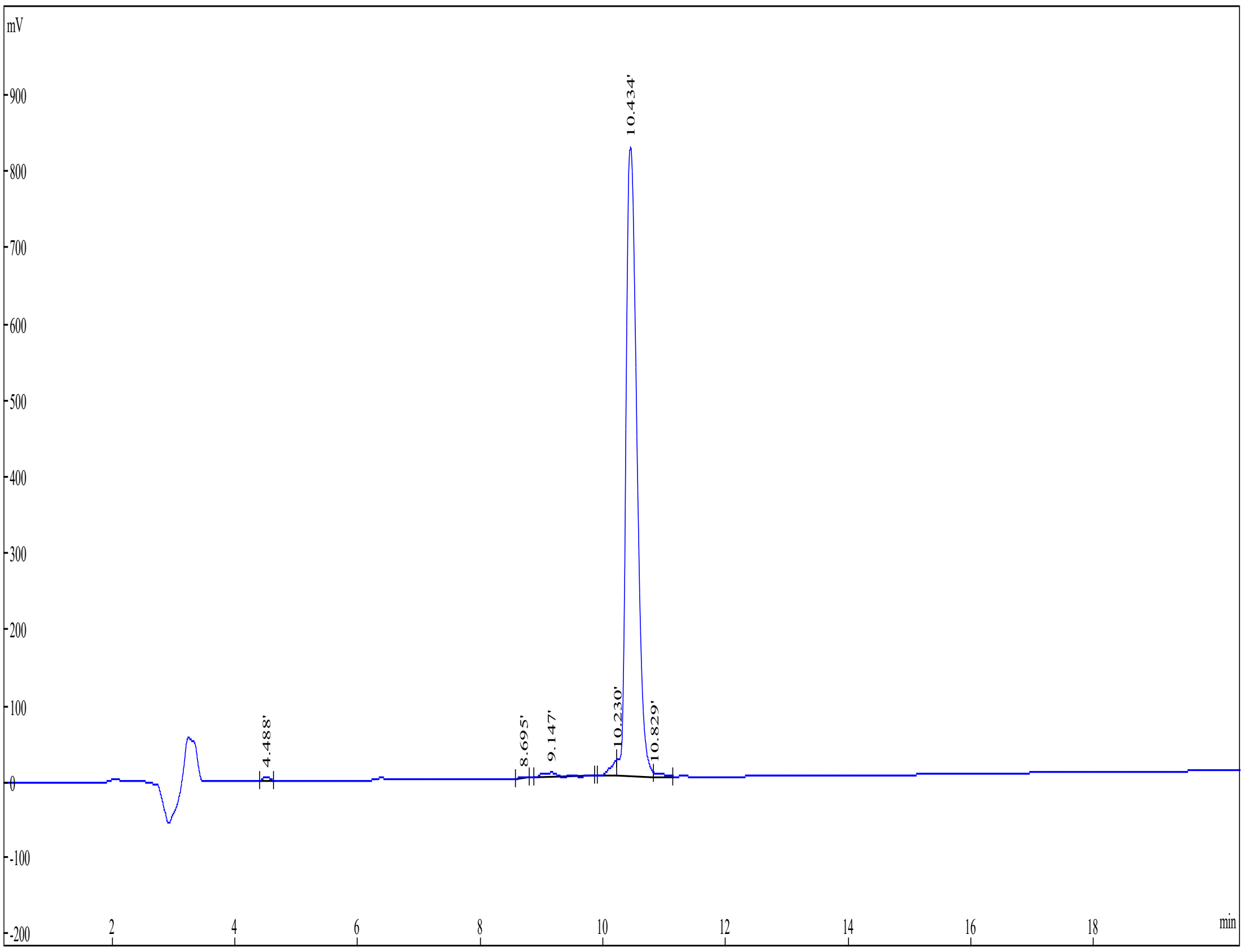

Fig. S2. (C) HPLC chromatogram of C9-P7. HPLC analysis was performed using a Kromasil 100-5C18 column (4.6 mm × 250 mm, 5 µm) at 25 °C with detection at 220 nm, a flow rate of 1.0 mL/min, and an injection volume of 10 µL. Mobile phase A consisted of 0.1% TFA in acetonitrile, while mobile phase B consisted of 0.1% TFA in water. Linear gradient: 35% A/65% B at 0.0 min, 60% A/40% B at 20.0 min, and 100% A/0% B at 20.1 min; total run time 20 min.

(D)

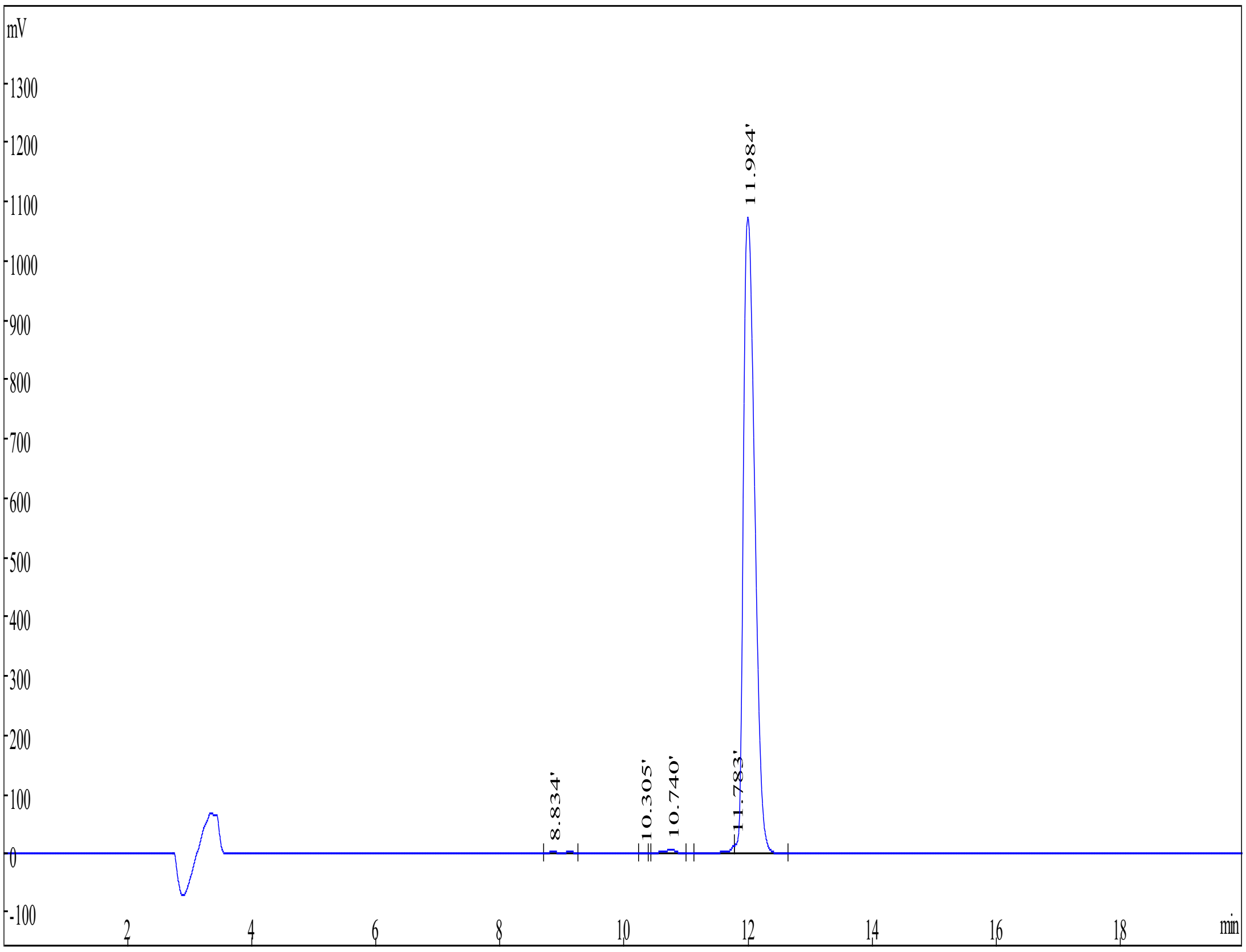

Fig. S2. (D) HPLC chromatogram of C10-P7. HPLC analysis was performed using a Kromasil 100-5C18 column (4.6 mm × 250 mm, 5 µm) at 25 °C with detection at 220 nm, a flow rate of 1.0 mL/min, and an injection volume of 10 µL. Mobile phase A consisted of 0.1% TFA in acetonitrile, while mobile phase B consisted of 0.1% TFA in water. Linear gradient: 35% A/65% B at 0.0 min, 60% A/40% B at 20.0 min, and 100% A/0% B at 20.1 min; total run time 20 min.
